# Fine-tuning of the setting of critical day length by two casein kinases in rice photoperiodic flowering

**DOI:** 10.1093/jxb/erx412

**Published:** 2017-12-10

**Authors:** Yasue Nemoto, Kiyosumi Hori, Takeshi Izawa

**Affiliations:** 1 Functional Plant Research Unit, National Institute of Agrobiological Sciences, Tsukuba, Japan; 2 Rice Applied Genomics Research Unit, National Institute of Agrobiological Sciences, Tsukuba, Japan; 3 Institute of Crop Science, National Agriculture and Food Research Organization, Tsukuba, Japan; 4 University of Tokyo, Faculty of Agriculture, Laboratory of Plant Genetics and Breeding, Bunkyo-ku, Tokyo, Japan

**Keywords:** Casein kinase, critical day-length, photoperiodic flowering, rice, short-day plants

## Abstract

Many short-day plants have a critical day length that fixes the schedule for flowering time, limiting the range of natural growth habitats (or growth and cultivation areas). Thus, fine-tuning of the critical day-length setting in photoperiodic flowering determines ecological niches within latitudinal clines; however, little is known about the molecular mechanisms controlling the fine-tuning of the critical day-length setting in plants. Previously, we determined that florigen genes are regulated by day length, and identified several key genes involved in setting the critical day length in rice. Using a set of chromosomal segment substitution lines with the genetic background of an elite *temperate japonica* cultivar, we performed a series of expression analyses of flowering-time genes to identify those responsible for setting the critical day-length in rice. Here, we identified two casein kinase genes, *Hd16* and *Hd6*, which modulate the expression of florigen genes within certain restricted ranges of photoperiod, thereby fine-tuning the critical day length. In addition, we determined that *Hd16* functions as an enhancer of the bifunctional action of *Hd1* (the Arabidopsis *CONSTANS* ortholog) in rice. Utilization of the natural variation in *Hd16* and *Hd6* was only found among *temperate japonica* cultivars adapted to northern areas. Therefore, this fine-tuning of the setting of the critical day length may contribute to the potential northward expansion of rice cultivation areas.

## Teaser

Two casein kinase genes, *Hd16* and *Hd6*, modulate florigen genes within certain restricted ranges of photoperiod, thereby fine-tuning the critical day length in rice and potentially determining ecological niches within latitudinal clines.

## Introduction

Floral transition, the major developmental switch from the vegetative to reproductive phase in plants, is regulated by both endogenous and environmental signals. Photoperiodic flowering, one of the most important biological systems in controlling floral transition, is regulated by light signals and the plant’s endogenous circadian rhythm (Thomas and Vince-Prue, 1997). Rice photoperiodic flowering has been investigated extensively as a model system of short-day (SD) plants. The rice florigen gene *Heading date 3a* (*Hd3a*) is regulated according to the recognition of critical day length, and several key genes have been identified that are necessary for setting this day length (Itoh *et al.*, 2010).

The critical day length determines whether a plant will flower under certain cultivation environments. For example, several rice cultivars usually grown in tropical areas fail to flower in temperate areas, as it becomes too cold to differentiate the panicles at the apex when day length falls below the critical level. Thus, the genetic setting of critical day length may be a major factor determining the range of cultivation for rice cultivars. Natural habitats of various Japanese duckweed (*Lemna*) accessions are distributed latitudinally, showing associations with their critical day length (Yukawa and Takimoto, 1976; Beppu and Takimoto, 1981). However, the molecular genetics underlying the fine-tuning of critical day length has yet to be elucidated. In rice, *Hd3a* gene expression was found to be affected by a single long-day (LD) stimulus, and the resulting change in *Hd3a* produced late flowering (Ishikawa *et al.*, 2011). Two distinct circadian-clock gates controlling the *Ehd1* floral activator and the *Ghd7* floral repressor sensitively and accurately regulate the threshold of *Hd3a* expression (Itoh *et al.*, 2010). Recently, we reported that the formation of a transcriptional complex containing both *Hd1* and *Ghd7* proteins could repress *Ehd1* expression under LD conditions (Nemoto *et al.*, 2016).

In rice, many quantitative trait loci (QTLs) have been implicated in regulating flowering time under field conditions, and positional cloning has been used to isolate the corresponding genes or functional nucleotide polymorphisms (Matsubara *et al.*, 2014). QTL analyses for flowering-time (or heading date) conducted on two temperate *japonica* cultivars, ‘Nipponbare’ and ‘Koshihikari’, revealed the involvement of two alleles, *Hd16* and *Hd17* (Matsubara *et al.*, 2008), and the causative genes were also identified (Matsubara *et al.*, 2014). *Hd16* and *Hd17* encode a casein kinase I (CKI) and an ortholog of Arabidopsis *EARLY FLOWERING 3* (*ELF3*), respectively (Matsubara *et al.*, 2012; Hori *et al.*, 2013). The Koshihikari allele of *Hd16* promotes flowering under natural conditions and decreases the kinase activity in comparison with the Nipponbare allele (Hori *et al.*, 2013). Because *Hd16* does not affect the expression pattern of clock-associated genes, it might control flowering time in the photoperiodic pathway without affecting circadian rhythms (Hori *et al.*, 2013). CKI is a member of the highly conserved serine/threonine-specific casein kinases that are related to various signal-transduction processes in eukaryotes (Cheong and Virshup, 2011). The rice CKI gene positively regulates brassinosteroid signaling (Liu *et al.*, 2003) and exerts negative regulation on gibberellin signaling (Dai and Xue, 2010). Moreover, it is reported that the *Hd16*/*EL1* (Kwon *et al.*, 2014) protein can phosphorylate a floral repressor, *Ghd7*, *in vitro* (Hori *et al.*, 2013). Another casein kinase gene, *Hd6*, encoding the CKII α subunit (Takahashi *et al.*, 2001), was also detected as a flowering-time QTL between the temperate *japonica* cultivar Nipponbare and an *indica* cultivar ‘Kasalath’ (reviewed by Hori *et al.*, 2016). CKII is implicated in multiple processes related to development and stress-responses including the circadian clock in plants; however, it belongs to a serine/threonine kinase family that is evolutionally distinct from CKI (Mulekar and Huq, 2014). Although *Hd6* is also not involved in the circadian rhythm, it delays flowering under LD conditions only in the presence of functional *Hd1* (Ogiso *et al.*, 2010). Interestingly, the *Hd6* gene product does not interact with or phosphorylate the *Hd1* gene product *in vitro* (Ogiso *et al.*, 2010). It has been reported that *Hd16* and *Hd6* gene products interacted with the *Hd2*/*OsPRR37* protein *in vivo*, and phosphorylated different regions of this protein *in vitro* (Kwon *et al.*, 2015). *Hd2* has been detected previously in an F_2_ population derived from a cross between Nipponbare and Kasalath (Yano *et al.*, 1997; Yamamoto *et al.*, 1998) and isolated as the *OsPRR37* gene, an Arabidopsis TOC1 homolog (or a pseudo-response regulator gene) (Koo *et al.*, 2013).

Recently, a genetic resource termed ‘chromosomal segment substitution lines’ (CSSLs) has been developed in rice for detection of QTLs with small effects, and is a set of genetic lines that have distinct genomic fragments introgressed from a recurrent cultivar into a background parent cultivar so as to span the entire region of the genome with the introgressed fragments (Ebitani *et al.*, 2005, Keurentjes *et al.*, 2007). An *indica* cultivar, ‘Nona Bokra’, showed extremely late flowering compared to the *japonica* cultivar Koshihikari under LD conditions (Uga *et al.*, 2007). CSSLs with Nona Bokra as the donor and Koshihikari as the recipient background cultivar were developed and QTL analysis was performed for flowering time in the field in Tsukuba, Japan (Takai *et al.*, 2007). Although several flowering-time QTLs have been detected and candidate genes have been proposed for a few of them, it has so far been experimentally confirmed that Nona Bokra has a defective allele of one of the florigen genes, *RFT1*, due to an amino acid substitution (Ogiso-Tanaka *et al.*, 2013).

 In this study, using further analysis of the Nona Bokra–Koshihikari CSSLs and a set of newly developed nearly isogenic lines (NILs) of the *Hd6* and *Hd16* genes, we demonstrate that the fine-tuning of critical day length is set by these two CK genes. Mutations in the CK genes has no effect on the rice circadian clocks. Furthermore, we show that *Hd6* and *Hd16* are involved in the actions of both *Hd1* and *Ghd7* to control *Ehd1*, *Hd3a*, and *RFT1*.Our results suggest that this fine-tuning of the setting of the critical day length by natural variation in *Hd6* and *Hd16* may contribute to a potential northward expansion of rice cultivation areas.

## Materials and methods

### Plant material and growth conditions

We used CSSLs derived from a cross between the *Oryza sativa temperate japonica* cultivar Koshihikari as the recipient and the *indica* cultivar Nona Bokra as the donor produced by Takai *et al.* (2007). A set of four new NILs for *Hd16* and *Hd6* in the genetic background of Koshihikari were developed, including NILs for *Hd16* with the functional allele of *Hd6*, by crossing ‘Kanto IL5’ by marker-assisted selection (see Supplementary Fig. S1 at *JXB* online; Hori *et al.*, 2013). Kanto IL5 is one of the isogenic lines derived from crosses between Koshihikari and Kasalath. It has a 170-kb segment of the Kasalath chromosome around the *Hd6* allele in the Koshihikari background, which means that Kanto IL5 has a functional *Hd6* allele of Kasalath (Takeuchi, 2011). We were able to guarantee that the genomic fragment has a single gene mutation to affect flowering-time since this Kasalath fragment had been fine-mapped when these genes were cloned (Takahashi *et al.*, 2001; Hori *et al.*, 2013). In the present work, we used Koshihikari as the background cultivar. *Hd16* was originally cloned as a flowering-time QTL between Nipponbare and Koshihikari (Hori *et al.*, 2013). Furthermore, there is no QTL at the *Hd6* locus between Nipponbare and Koshihikari, indicating that Koshihikari has the same defective allele of *Hd6* as that of Nipponbare. Taken together, it has been proved that the four NILs used in this work have only the *Hd6* and *Hd16* genes to cause differences in flowering-time. We also used a set of NILs for *Hd16* and *Hd1* described in Hori *et al.* (2013). Plants were grown in a growth chamber under various photoperiod conditions (10, 12, 12.5, 13, 13.5, or 14.5 h); light was provided by metal halide lamps (ca. 450 μmol m^–2^ s^–1^). Temperature was maintained at 28/24 °C according to the day–night cycle. Light pulses in night-break experiments were provided using a red LED panel (intensity of 12.5 μmol m^–2^ s^–1^) for 15 min at the mid-point of the dark period. The flowering time was recorded as the time (or date) when the first panicle emerged.

### Expression and sequencing analyses

Total RNA was extracted from leaves using TRIZOL (Invitrogen) according to the manufacturer’s instructions. cDNA was synthesized after treatment with DNase I (Nippongene) from 5 μg of total RNA using Superscript II RTase (Invitrogen). Realtime qRT-PCR was carried out using the TaqMan PCR protocol (Nippongene) or the Power SYBR Green protocol (ABI) on an ABI PRISM 7900 Sequence Detection System according to the manufacturer’s instructions. The gene-specific primers and TaqMan probe sequences were as described previously (Nemoto *et al.*, 2016). A rice ubiquitin gene (*Os02g0161900*) was used for normalization. *Hd2* gene expression was analysed, and the PCR primers and TaqMan probes were as follows: *Hd2*-F, 5′-CAGAAAAGGAAAGAGCGCAAC-3′; *Hd2*-R, 5′-CTGCTCGGCCAGCCTC-3′; TaqMan probe, 5′-TCGGAAAAAAGGTGCGGTACCAGAG-3′. We determined the sequences of the PCR products of *Hd16* and *Hd6* expressed in Nona Bokra cDNA by using primers designed from Nipponbare and Kasalath cDNA sequences, respectively (see Supplementary Fig. S2).

## Results

### The essential genes for flowering under LD conditions in Nona Bokra are located on chromosomes 3 and 6

**Fig. 1.**
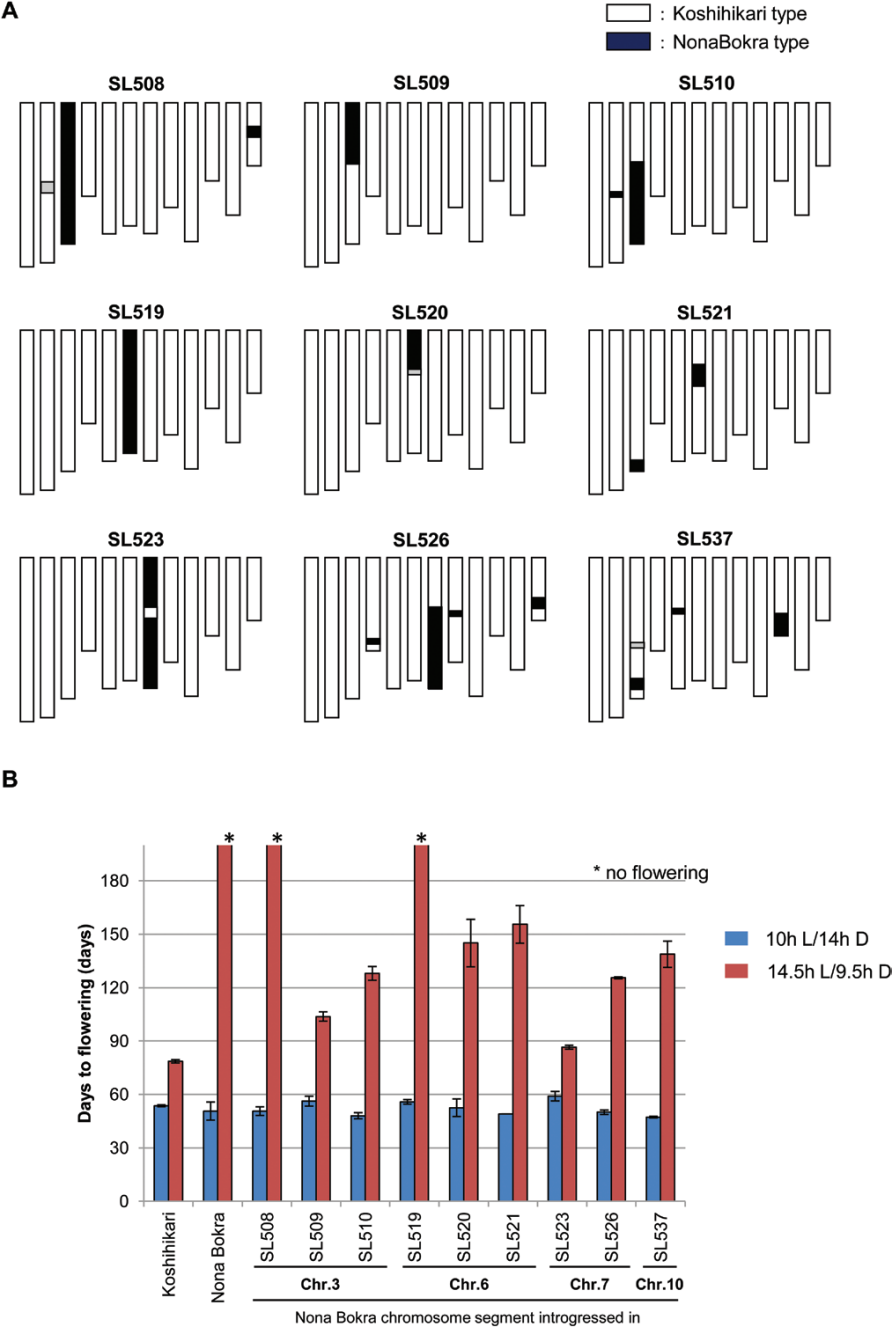
Graphical representations of genotypes (A), and flowering phenotypes (B) of nine chromosomal segment substitution lines (CSSLs). (A) Graphical illustration of the genotypes of nine CSSLs. The black and white regions denote homozygous Nona Bokra and Koshihikari alleles, respectively. The gray regions indicate heterozygous alleles. (B) The flowering phenotype with their recurrent and donor parents, Koshihikari and Nona Bokra, respectively, under 10 h L/14 h D or 14.5 h L/9.5 h D conditions. Data are the mean value of flowering time with ±SD (*n*=5). Asterisks indicate plants with no flowering up to 200 d after sowing.

In this study, we used a subset of CSSLs carrying chromosomal segments from Nona Bokra in the Koshihikari genetic background, which were developed by Takai *et al.* (2007). First, we selected nine CSSLs that exhibited a severe late-flowering phenotype under natural field conditions in Tsukuba (36 °N; Takai *et al.*, 2007). Compared with previous studies using populations of a cross between Nipponbare and Kasalath (reviewed by Yano *et al.*, 2001), the selected CSSLs have introgressed fragments located on chromosome 3 (*Hd9*, *Hd8*, and *Hd6*), chromosome 6 (*Hd3a*, *Hd3b*, and *Hd1*), chromosome 7 (*Hd4* and *Hd2*), and chromosome 10 (*Hd14*) (Fig. 1A, Yano *et al.*, 2001). Then, we grew the CSSLs under 10 h light (L)/14 h dark (D) and 14.5 h L/9.5 h D conditions to examine their flowering times (Fig. 1B). Under 10 h L/14 h D conditions, all lines flowered at ~50 d after sowing, with no significant differences in flowering time among the recurrent parents (Koshihikari and Nona Bokra types) and the CSSLs tested (Fig. 1B).

Under the 14.5 h L/9.5 h D conditions, Nona Bokra showed no flowering during the test period, whereas Koshihikari flowered at ~78 d after sowing. All the tested CSSLs exhibited significantly later flowering than that of Koshihikari (Fig. 1B). We found that two CSSLs, SL508 and SL519, were similar to Nona Bokra, as they showed no flowering under 14.5 h L/9.5 h D conditions up to 200 d after sowing. The majority of chromosomes 3 and 6 were substituted with Nona Bokra chromosomal segments in SL508 and SL519, respectively (Fig. 1A). This result suggested that the essential genes for the non-flowering phenotype under 14.5 h L/9.5 h D conditions are located independently on chromosomes 3 and 6 of Nona Bokra.

The rice florigen gene *RFT1*, located on chromosome 6, is a major floral activator under 14.5 h L/9.5 h D conditions (Komiya *et al.*, 2009). Nona Bokra has a defective allele of *RFT1*; therefore, only *Hd3a* functions as a florigen in this variety (Ogiso-Tanaka *et al.*, 2013). As SL519 has a Nona Bokra-type chromosome 6, it possesses non-functional *RFT1*. Although SL520 also has non-functional *RFT1* (Ogiso-Tanaka *et al.*, 2013), it flowered at ~150 d after sowing under 14.5 h L/9.5 h D conditions (Fig. 1B). This result was consistent with the fact that *RFT1* RNAi plants exhibit delayed flowering under the same photoperiod conditions (Komiya *et al.*, 2009), implying that the other florigen gene, *Hd3a*, might not function appropriately in SL519. Therefore, some unknown but critical natural variation inhibiting the activity of *Hd3a* might exist in the Nona Bokra segment in SL519. Although many of the Nona Bokra-type chromosomal segments described above contain previously identified flowering-time QTLs such as *Hd2* on chromosome 7, no molecular analyses at the level of nucleotide polymorphism have been performed except for the *RFT1* gene (Ogiso-Tanaka *et al.*, 2013).

### Key QTLs for recognition of critical day length are located on the long arm of chromosome 3 in Nona Bokra

**Fig. 2.**
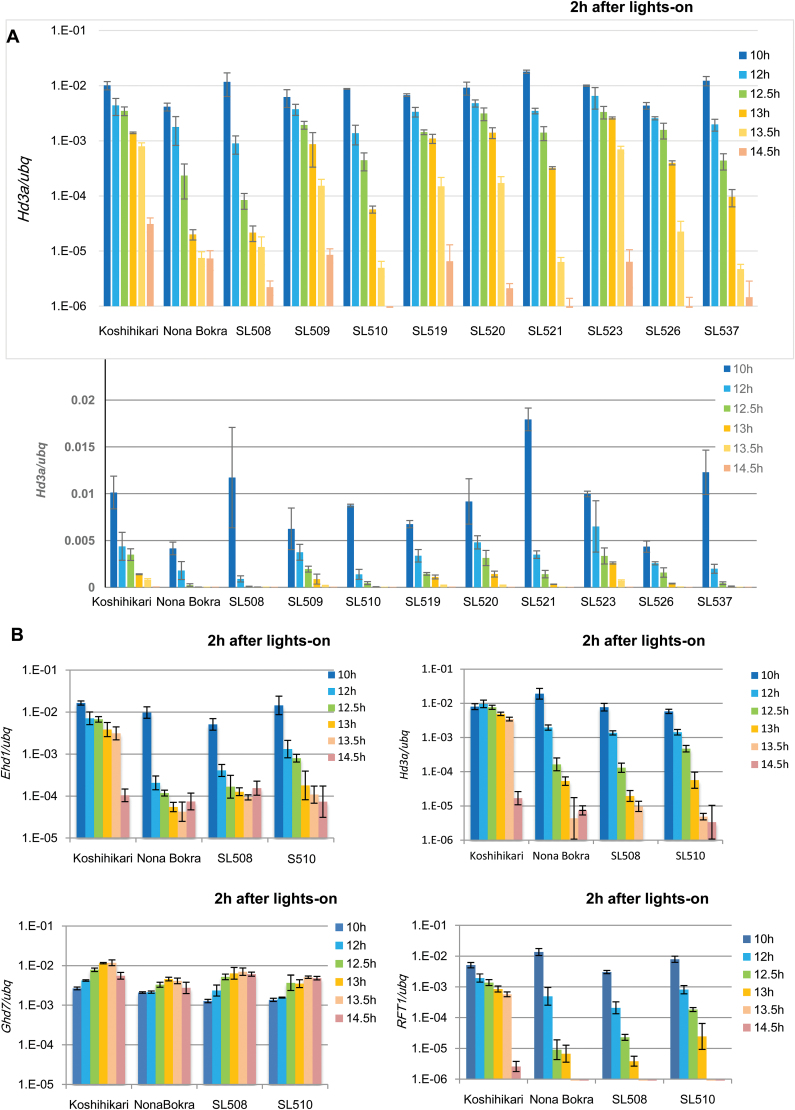
Expression analysis of CSSLs and their parents under various photoperiods. After a 6-d entrainment period, the fully expanded leaves were harvested from three replicates composed of two or three independent plants at 2 h after lights-on. (A) Expression of *Hd3a* under different photoperiods of nine CSSLs and their parents. The level of gene expression is shown on a normal (upper) and a log scale (lower). (B) The CSSLs were selecting according to the contribution of chromosome 3 from Nona Bokra and the expression patterns of *Ehd1*, *Hd3a*, and *RFT1* that were similar to that of Nona Bokra. Each value is the average of three biological replicates (means ±SD).

To examine the effect of Nona Bokra alleles on the setting of the critical day length, we examined *Hd3a* expression in selected CSSLs and the Koshihikari and Nona Bokra parents in the morning (i.e. 2 h after lights on) after a 6-d entrainment period under various day-length conditions (Fig. 2A). We found that the photoperiodic profile of *Hd3a* expression differed markedly in the Koshihikari and Nona Bokra types, with high expression levels of *Hd3a* being detected under 10 h L/14 h D conditions, whereas low levels were observed under 14.5 h L/9.5 h D conditions for all the tested lines. However, the pattern of the decrease in rate of *Hd3a* expression in relation to increasing photoperiod differed significantly in Koshihikari, Nona Bokra, and the tested CSSLs. We plotted these data with both logarithmic and normal axes (Fig. 2A). The peak values of *Hd3a* expression under 10 h L/14 h D conditions displayed similar ranges among the tested lines, and gene expression showed dynamic ranges of more than three orders of magnitude. In the case of Koshihikari, the expression level decreased gradually across photoperiods of 10−13.5 h but rapidly between photoperiods of 13.5 and 14.5 h. On the other hand, expression in Nona Bokra decreased proportionately photoperiods of 10−14.5 h. The expression level of *Hd3a* under 14.5 h L/9.5 h D conditions was ~10^−5^−10^−6^, which is around the detection limits of this quantitative RT-PCR analysis (Fig. 2A). The decreasing profiles of the tested CSSL lines could be classified into three patterns: the lines SL509, SL519, SL520, and SL523 belonged to the Koshihikari type; the lines SL508, SL510, and SL537 belonged to the Nona Bokra type; and the lines SL521 and SL526 exhibited intermediate patterns between the Koshihikari and Nona Bokra types. The SL508 and SL510 lines, which contained genomic fragments of chromosome 3 from Nona Bokra, exhibited effects that were as strong as those of the Nona Bokra type, and the two previously identified flowering-time QTLs, *Hd6* and *Hd16*, were located on the introgressed fragments of these two lines; therefore, we focused on SL508 and SL510 in this study. There is no candidate gene isolated yet for SL537.

We compared the related flowering-time gene expression patterns in SL508, SL510, and their donors (Fig. 2B). SL508 and SL510 exhibited gene expression of *Ehd1*, *Hd3a*, *Ghd7*, and *RFT1* that was similar to that of Nona Bokra (Fig. 2B). SL508 contains the majority of chromosome 3 from Nona Bokra (Fig. 1A), whereas SL510 contains only the lower part of chromosome 3 from this variety. QTL analyses of heading time using an F_2_ population derived from a cross between the Koshihikari and Nona Bokra types (Uga *et al.*, 2007) demonstrated that the candidate QTL contained the *Hd6* region on the lower part of chromosome 3. *Hd6*, originally identified as a QTL using the Nipponbare and Kasalath cultivars, encodes the alpha subunit of CK II. Nipponbare contains the non-functional allele, *hd6*, due to a non-synonymous change, whereas Kasalath contains a functional *Hd6* allele (Takahashi *et al.*, 2001). Koshihikari has the same non-functional *Hd6* allele as Nipponbare, whereas Nona Bokra might have a functional *Hd6* allele, similar to the Kasalath type (Uga *et al.*, 2007). The *Hd16* QTL for heading time, which is in the vicinity of *Hd6* on chromosome 3, was detected in a cross between the Nipponbare and Koshihikari types (Matsubara *et al.*, 2008). *Hd16* was shown by map-based cloning to encode a casein kinase-I protein (Hori *et al.*, 2013). Thus, we focused on *Hd16* and *Hd6* as candidate fine-tuning genes for setting the critical day length for *Hd3a* expression in rice. Since it is already known that *Hd6* and *Hd16* in Koshihikari have defective mutations (Takahashi *et al.*, 2001; Hori *et al.*, 2013), in this current work we sequenced these genes in Nona Bokra and found that both of them appear to be the functional alleles (see Supplementary Fig. S2).

### Casein kinase I and II are involved in setting the genes for critical day length

**Fig. 3.**
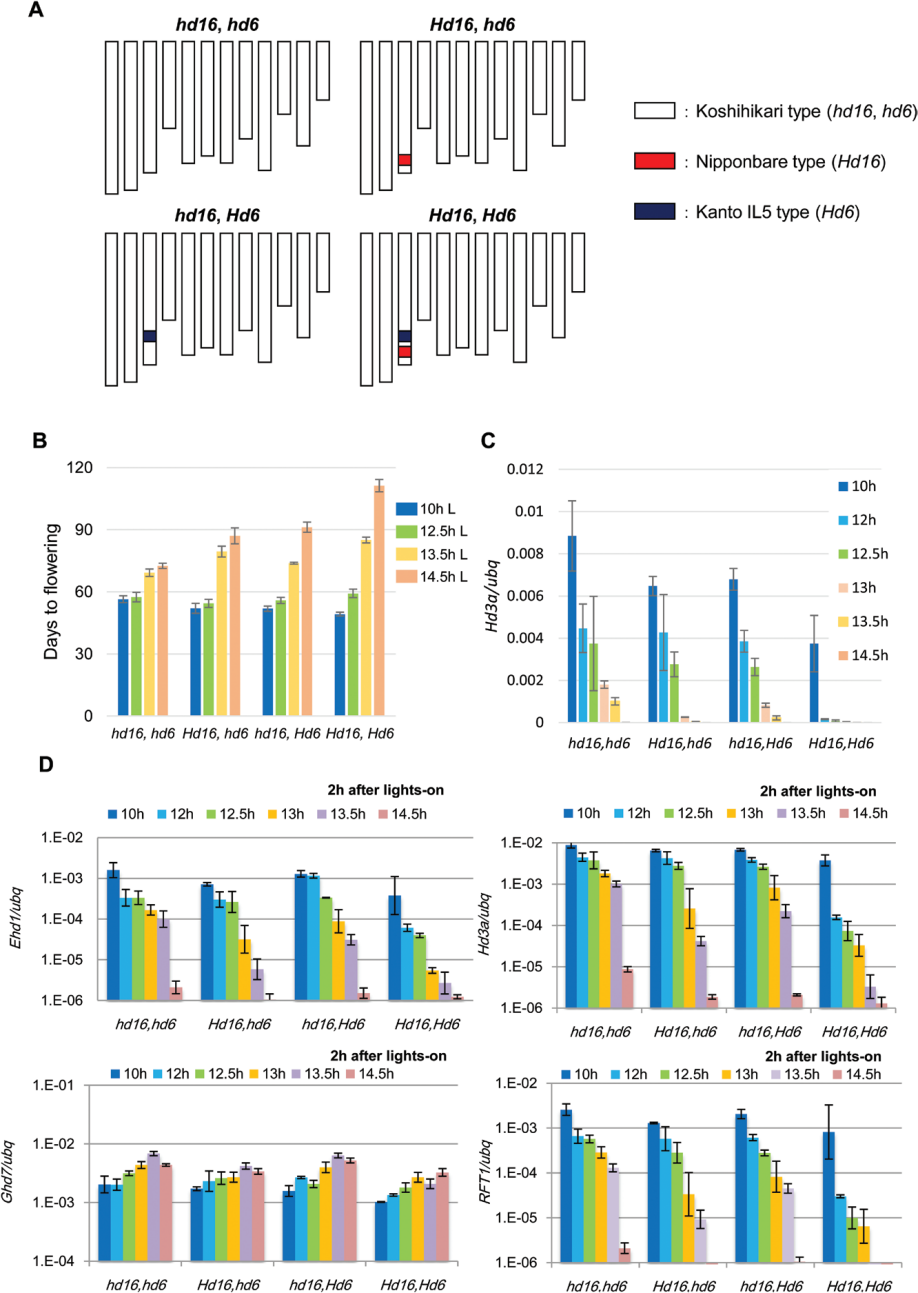
Comparison of flowering time and photoperiodic response in NILs of Koshihikari. (A) Graphical representation of the NIL genotypes. Functional *Hd16* and *Hd6* were introduced from Nipponbare and Kanto IL5, respectively. The *Hd6* allele of Kanto IL5 was originated from Kasalath (Takeuchi, 2011). (B) Days to flowering of plants grown under various photoperiods after sowing. Data are means ±SD for 6−10 plants per genotype. NIL genotypes are indicated. (C, D) Three-week-old NIL plants were entrained for 6 d with various photoperiods and expression patterns of *Hd3a*, *Ehd1*, *Ghd7*, and *RFT1* are shown. Data for *Hd3a* are shown on a normal scale (C) and on log scale (D). The experimental procedures were the same as those described in Fig. 2.

To elucidate the contribution of the *Hd16* and *Hd6* genes in setting the critical day length, we produced a series of four nearly isogenic lines (NILs) containing all possible combinations of the defective and functional alleles of *Hd16* and *Hd6* (originated from Nipponbare and Kasalath alleles, respectively) in the genetic background Koshihikari (Hori *et al.*, 2013; Fig. 3A). Notably, *Hd16* and *Hd6* are closely linked genetically, and the physical distance between the two genes is ~1.5 Mb. We investigated the effects of the *Hd16* and *Hd6* alleles on the flowering phenotype under 10-, 12.5-, 13.5-, and 14.5-h photoperiod conditions (Fig. 3B). Under 10-h photoperiod conditions, plants exhibited slightly earlier flowering when both the *Hd16* and *Hd6* alleles were functional; conversely, they displayed markedly late flowering under a 14.5-h photoperiod (Fig. 3B). Under 12.5 h L/11.5 h D conditions, all lines flowered at almost the same time, which was inconsistent with the gene expression patterns of related genes at their seedling stages (Fig. 3C, D). This result implied that the gene expression patterns at this developmental stage might not be correlated directly with their flowering time. Under 13.5 h L/9.5 h D conditions, late flowering was observed in plants with the functional allele of *Hd16*. Comparing the functions for the two alleles, *Hd16* had the stronger effect for flowering under this photoperiod. Under 14.5 h L/9.5 h D conditions, the functional alleles of both *Hd16* and *Hd6* contributed significantly to a delay in flowering, and each gene functioned additively to control flowering time (Fig. 3B). These results indicated that the functional alleles of *Hd16* and *Hd6* were related to both promotion and repression of flowering time under the 10- and 14.5-h photoperiods, respectively.

Next, we examined the gene expression pattern of *Hd3a* in the morning with varying day lengths (Fig. 3C). The results indicated that both *Hd6* and *Hd16* are major determinants of critical day length for *Hd3a* gene expression. We further examined the gene expression of related flowering-time genes (Fig. 3D). The expression patterns of *Ghd7* were similar among these lines, with expression increasing with longer photoperiods. Only the *Hd16Hd6* line displayed a Nona Bokra-type in the expression of *Ehd1*, *Hd3a*, and *RFT1*. *hd16Hd6* and *Hd16Hd6* showed intermediate patterns between Koshihikari and Nona Bokra. The *hd16hd6* line exhibited gene expression patterns of *Ehd1*, *Hd3a*, and *RFT1* that were similar to those of the Koshihikari type (Fig. 3D). These results indicated that the functional alleles of *Hd16* and *Hd6* that were necessary for the rapid decrease depended on longer photoperiods, in a manner similar to the Nona Bokra type. Significant repressions of *Hd3a* and *RFT1* gene expression were observed between the 13- and 13.5-h photoperiods, but not at the 10-, 12-, and 14.5-h ones, for the *hd16Hd6* and *Hd16hd6* lines; in contrast, significant repressions were observed at the 12.5-, 13-, and 13.5-h photoperiods, but not at 10- and 14.5-h ones for the *hd16hd6* line. These results indicated that the combination of *Hd16* and *Hd6* alleles fine-tuned the critical day length for florigen gene expression, which might determine appropriate cultivation areas by setting flowering times (or heading dates) under certain environments.

### The effects of *Hd16* and *Hd6* on diurnal expression patterns of flowering-related genes

**Fig. 4.**
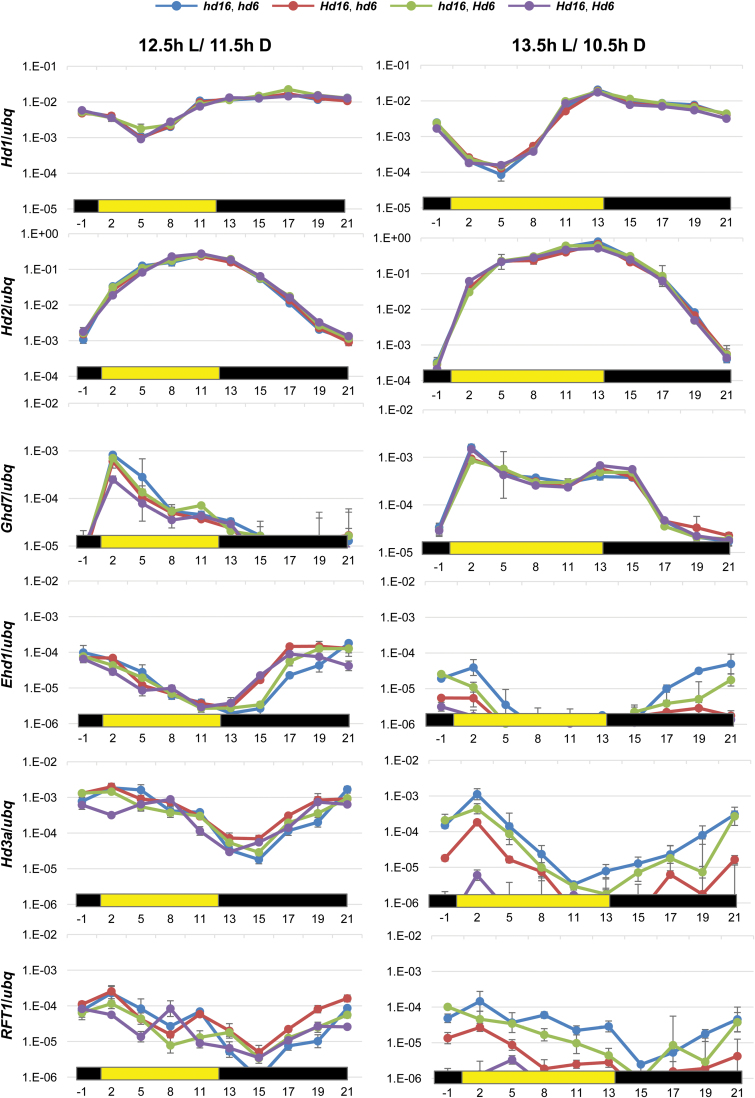
Diurnal expression patterns of flowering-time genes under 12.5 h L/11.5 h D (left) and 13.5 h L/10.5 h D (right) photoperiods in NILs of Koshihikari. Yellow and black horizontal bars represent light and dark periods, respectively. Leaves were collected every 2 or 3 h during light or dark conditions, respectively. Each value is the mean of three biological replicates consisting of two or three independent plants (means ±SD).

We examined the diurnal expression patterns of flowering-time genes at intervals of 2 h in the dark or 3 h in the light for one day using 3-week-old seedlings of NILs grown under conditions of 12.5 h L/11.5 h D (Fig. 4 left panel) and 13.5 h L/10.5 h D (Fig. 4 right panel). The diurnal expression patterns of *Hd1*, *Hd2*, and *Ghd7* were not affected by the *Hd16* or *Hd6* alleles. *Ehd1* expression was activated only with the *Hd16* functional allele during the early night period from 0.5 to 4.5 h after lights-off under 12.5 h L/11.5 h D conditions. Thus, *Hd16* could function as an *Ehd1* activator in the early night regardless of *Hd6* function. In addition, under 12.5 h L/11.5 h D conditions, the florigen genes *Hd3a* and *RFT1* expressed a similar diurnal pattern; however, the expression level of *Hd3a* was higher than that of *RFT1*. At 2 h after lights-on, *Hd3a* expression was slightly repressed by the functional alleles of both *Hd16* and *Hd6*. *RFT1* expression was also slightly repressed by *Hd16* and *Hd6* from 2 to 5 h after lights-on. Taking these findings together, under 12.5 h L/11.5 h D conditions, *Hd16* seems to have a dual function for flowering genes as a promoter at night (independent of *Hd6*) and as a repressor in the morning (dependent on *Hd6*). Under 10 h L/14 h D conditions, the expression levels of *Ehd1*, *Hd3a*, and *RFT1* were similar in the morning (Fig. 3D), but slightly lower only in the *Hd16Hd6* lines. We further examined the expression level of *Ehd1* at 1 h before and 5 h after lights-off (see Supplementary Fig. S3) under 10 h L/14 h D conditions, and observed activation of *Ehd1* by *Hd16*. Thus, *Hd16* might function as a floral promoter under 10 h L/14 h D conditions.

We next examined gene expression with NILs grown under 13.5 h L/10.5 h D conditions (Fig. 4 right panel). These genes were synergistically repressed by both *Hd16* and *Hd6*. The diurnal expression patterns of *Hd1*, *Hd2*, and *Ghd7* were not affected by *Hd16* or *Hd6*, and showed patterns similar to those observed under 12.5 h L/11.5 h D conditions. On the other hand, the gene expression levels of *Ehd1*, *Hd3a*, and *RFT1* were highly repressed by either *Hd16* or *Hd6* and by the combination of *Hd16* and *Hd6* (Fig. 4 right panel). In these experiments, *Hd16* had stronger repression activity than *Hd6* (Fig. 4 right panel). These results were roughly consistent with the flowering phenotypes, in that the functional *Hd16* caused a slight flowering delay under 13.5 h L/10.5 h D conditions (Fig. 3B).

### *Hd16* did not enhance *Ghd7* function induced by night-break under a 10-h photoperiod

**Fig. 5.**
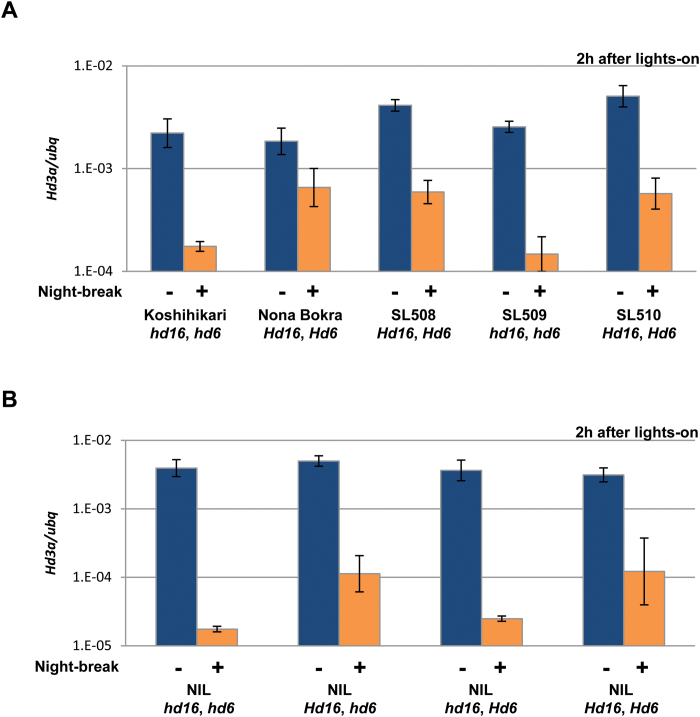
Effects of interrupting the dark period (night-break) on the expression of *Hd16* and *Hd6* alleles. CSSLs of Koshihikari (A) and NILs of Koshihikari (B) were tested. Each plant was grown under 10 h L/14 h D conditions for 14 d prior to the night-break treatments. Expression analyses were carried out the following day after 2 h of lights-on. Orange bars indicate night-break treatment (+) and blue bars indicate controls without a night break (–). Mean values ±SD were obtained from three biological repeats.

The Hd16 recombinant protein (rHd16) phosphorylates rGhd7 specifically *in vitro* (Hori *et al.*, 2013), and phosphorylation of *Ghd7* has been thought to enhance its repressive activity. To verify whether *Ghd7* repression activity was enhanced by *Hd16* through phosphorylation, we performed a series of night-break experiments to induce *Ghd7* transcription by introducing light pulses at night (Itoh *et al.*, 2010) in the related CSSL lines (Fig. 5A) and the *Hd16 Hd6* NILs (Fig. 5B). We then examined the repression level of *Hd3a* in the morning following the night-break treatment. We observed that the night-break effect for *Hd3a* was affected by the function of *Hd16*, but was not dependent on the *Hd6* genotype (Fig. 5B). After a night-break, the expression of *Hd3a* was lower in plants with the non-functional *hd16* allele than in those with the functional *Hd16* allele, as indicated by the fact that the night-break repression among the tested lines was clearest in the *hd16hd6* line. This finding was contrary to our prediction that the night-break-induced *Ghd7* gene product might be phosphorylated by *Hd16*, i.e. that its repression activity might be enhanced with the functional *Hd16* allele. An investigation of the night-break effect on the CSSLs and their donors (Fig. 5A) showed results that were consistent with those found in the NILs. The *Hd3a* expression level after a night-break was more repressed with the non-functional *hd16* allele than with the functional *Hd16* allele. Therefore, we propose that *Hd16* may function as a floral promoter under 10 h L/14 h D conditions, independent from the *Ghd7* repressor function. The *Ghd7* gene product induced by a night-break under 10 h L/14 h D conditions might not be phosphorylated *in vivo* by *Hd16*. Alternatively, the phosphorylated *Ghd7* gene product might not function as a repressor. Taken together, our results indicate that *Hd16* is unrelated to the night-break repression mediated by *Ghd7*, and it can induce *Hd3a* and *RFT1* independently at night under 10 h L/14 h D conditions.

### *Hd16* seems to enhance *Hd1* activator and repressor functions under SD and LD conditions, respectively

**Fig. 6.**
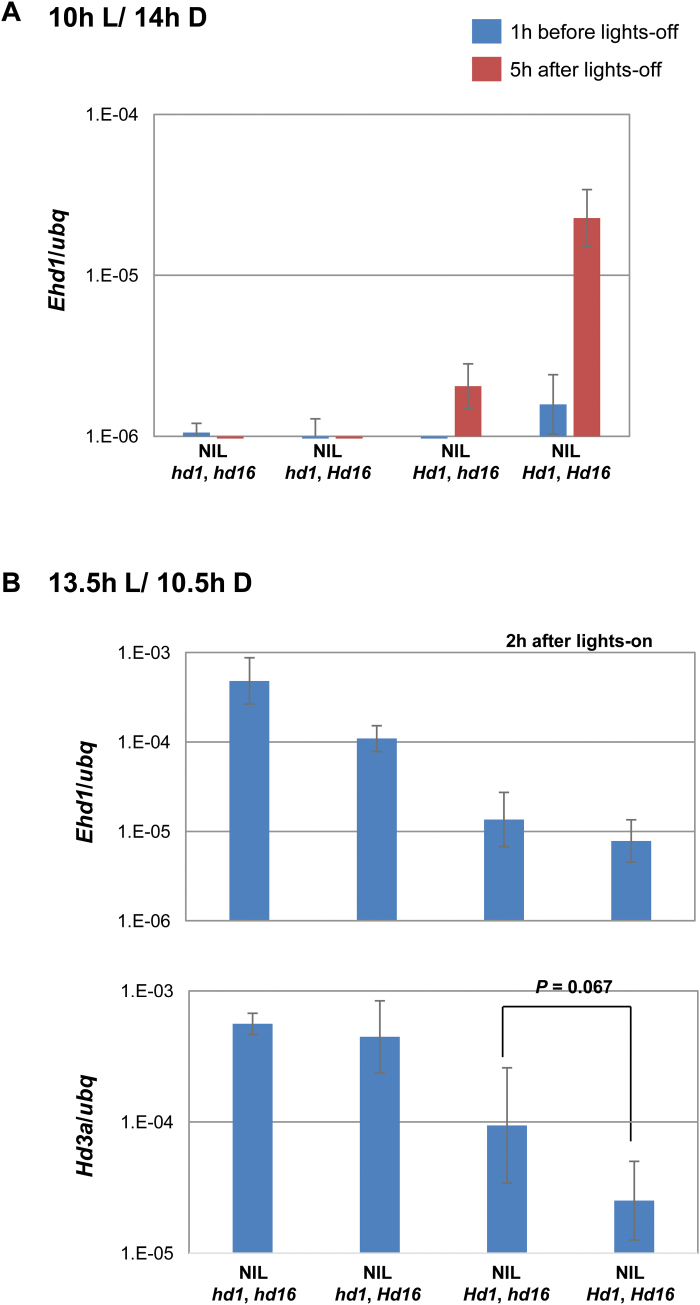
*Hd16* enhanced *Hd1* activity under both (A) 10 h L/14h D and (B) 13.5 h L/10.5 h D conditions in Koshihikari background NILs for the *Hd16* and *Hd1* alleles. (A) *Ehd1* expression levels were compared in *Hd16* and *Hd1* NILs. Leaves were harvested 1 h before and 5 h after lights-off under 10 h L/14 h D conditions. (B) *Ehd1* and *Hd3a* expression levels were compared 2 h after lights-on under 13.5 h L/10.5 h D conditions. The repression of *Hd3a* by *Hd16* with the functional allele of *Hd1* showed some statistical significance (one-tailed *t*-test, *P*=0.067). Mean values ±SD were obtained from three biological repeats.

Previously, we reported that *Ehd1* was activated by *Hd1* early during the night period under 10 h L/14 h D conditions (Nemoto *et al.*, 2016). In the present study, we found that *Ehd1* was activated by *Hd16* early during the night under 12.5-h (Fig. 4 left panel) and 10-h (see Supplementary Fig. S3) photoperiod conditions. These findings led us to investigate whether *Hd16* was required for *Ehd1* activation by *Hd1*. Using a series of NILs for the *Hd1* and *Hd16* alleles in the Koshihikari genetic background produced by Hori *et al.* (2013), we assessed the expression levels of *Ehd1* before and after lights-off under 10 h L/14 h D conditions. High levels of *Ehd1* transcript were observed at 5 h after lights-off in the *Hd1Hd16* line, while low levels were found in the other lines (Fig. 6A). This result indicated that *Ehd1* activation by *Hd1* requires *Hd16* function. *Hd1* has a bifunctional role for flowering, which is switched by differing photoperiods: it functions as a floral promoter under SD conditions and as a floral repressor under LD conditions. Thus, we further examined whether the repression activity of *Hd1* required *Hd16* under 14.5 h L/9.5 h D conditions. NILs on *Hd1Hd16* were grown under 13.5 h L/10.5 h D conditions for 3 weeks and then examined for gene expression of *Ehd1* and *Hd3a* at 2 h after lights-on (Fig. 6B). The expression levels of *Ehd1* and *Hd3a* were repressed by *Hd1*. Under 13.5 h L/9.5 h D conditions, *Hd16* seemed to enhance *Hd3a* repression activity by *Hd1*. In addition, *Hd16* seemed to repress *Ehd1* in the presence of the defective *Hd1* allele. These results suggest that *Hd16* functions to enhance the *Hd1* repressor activity even under LD conditions. r*Hd1* protein is not phosphorylated by r*Hd16* protein (Hori *et al.*, 2013); thus, indirect mechanisms may be involved in the enhancement of *Hd1* repressor activity.

## Discussion

### QTLs essential for LD flowering are located on chromosomes 3 and 6 in Nona Bokra

The rice *indica* cultivar Nona Bokra, which originated in India, exhibits a strong photoperiodic response. QTL analyses for flowering time were carried out with an F_2_ population derived from a cross between Nona Bokra and Koshihikari (Uga *et al.*, 2007), and the CSSLs were derived from a backcross between Nona Bokra as the donor and Koshihikari as the recipient cultivar (Takai *et al.*, 2007). All QTLs with Nona Bokra alleles contributed to late flowering under natural field conditions in Tsukuba, Japan. Here, we study a selected set of the CSSLs under artificially controlled photoperiod conditions.

Two lines, SL508 and SL519, showed no flowering even at 180 d under 14.5 h L/9.5 h D conditions (Fig. 1B). A major QTL for late flowering was identified on SL519 as a natural variation in the *RFT1* gene (Ogiso-Tanaka *et al.*, 2013). Further analysis was needed on SL519, as SL508 contained the entire region of chromosome 3 from Nona Bokra (Fig. 1A). The flowering-time phenotypes of the related CSSLs SL509 and SL510, which each contained approximately half of the segments of chromosome 3, suggested that critical genetic interactions occur between distinct QTLs to confer non-flowering phenotypes under LD conditions.

This study focused on *Hd16* and *Hd6* located on chromosome 3, in which the background parent cultivar, Koshihikari, has both the defective alleles of *Hd6* and *Hd16*, and we analysed them using a series of newly developed NILs. We demonstrated that both *Hd16* and *Hd6* genes contributed to the fine-tuning of the setting of the critical day length (Fig. 3B–D). This is the first study to identify genetic determinants for changing the critical day length setting in photoperiodic flowering.

### *Hd16* and *Hd6* contribute to fine-tuning of the setting of the critical day length in rice photoperiodic flowering

The sensitive and accurate threshold control of *Hd3a* expression to set the critical day length in rice is controlled by two distinct gating mechanisms through *Ehd1* and *Ghd7* (Itoh *et al.*, 2010). The fine-tuning of the setting of the critical day length in *Lemna* flowering may determine its natural growth habitats (Yukawa and Takimoto, 1976; Beppu and Takimoto, 1981). Thus, it is possible that fine-tuning systems are required for setting the critical day length for florigen expression in natural variants of rice. The critical day length is apparently determined by both the expression levels of florigen genes in the leaves and the sensitivity for florigen proteins at the apex region. It has been reported that young rice seedlings can clearly induce expression of the *Ehd1*, *Hd3a*, and *RFT1* genes according to the day lengths to which plants are subjected, but the expression of these florigen genes at the developmental stages is not able to determine flowering-time, perhaps due to immature perception systems for the florigen proteins at the apex, and/or low peak levels of florigen gene expression. The threshold of florigen gene expression required to induce floral transition depends on the developmental stage at which the plants are tested, and then for a given stage we can experimentally determine the critical day length. Expression levels of florigen genes even at the young developmental stage were critically regulated by day length, suggesting that plants at this stage can be targeted to elucidate critical day-length recognition in terms of gene regulation. In this study, we found that *Hd16* and *Hd6* contribute to the fine-tuning of the setting of this critical day length for florigen gene expression in rice. *Hd16* and *Hd6* encode CKI and CKII, respectively, which are members of an evolutionally conserved serine/threonine kinase family. *Hd16* and *Hd6* are not involved in the circadian clock system in rice (Ogiso *et al.*, 2010; Hori *et al.*, 2013). It is still possible that phosphorylation of target transcriptional protein complexes not related to circadian clock systems may enhance the transcriptional activation and/or repression of florigen genes.

**Fig. 7.**
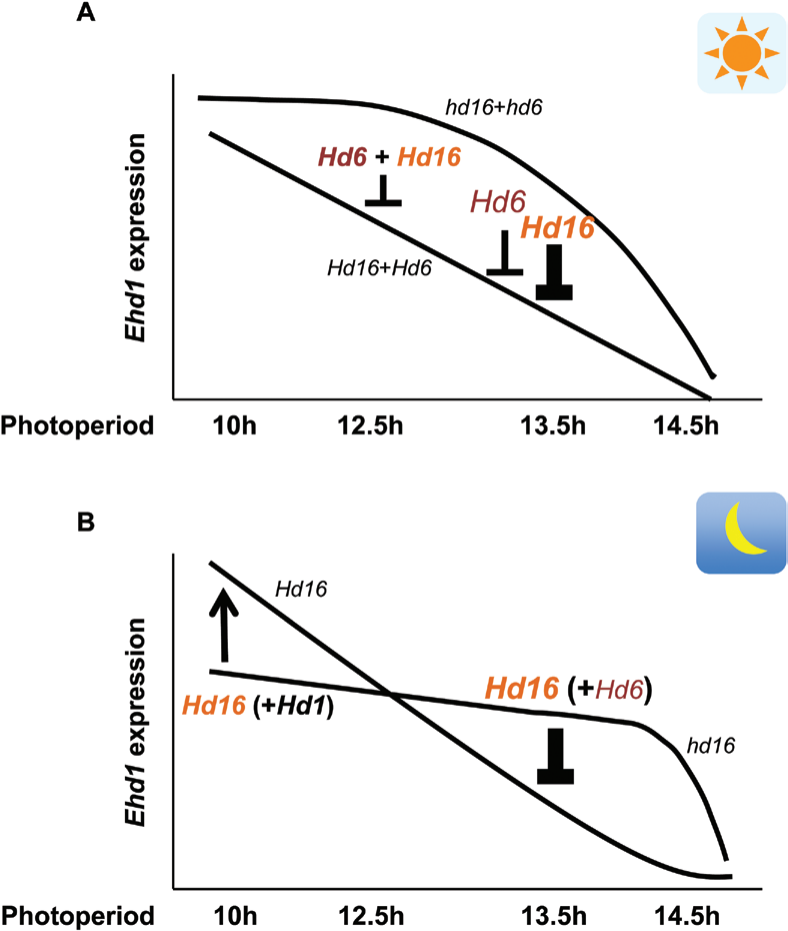
Illustration of *Ehd1* expression regulated by *Hd6* and *Hd16* in the morning (A) and at night (B) under various photoperiods. The *y*-axis indicates the expression level of *Ehd1*, and the *x*-axis shows the photoperiod. We compared functional (*Hd16* and *Hd6*) and non-functional (*hd16* and *hd6*) alleles in the morning (A). Under a 12.5-h photoperiod, *Hd16* and *Hd6* function together as a repressor. As the day length increases, they show repressor activity independent of each other; *Hd16* shows higher repressor activity than *Hd6*. (B) At the beginning of the night, *Hd1* activates *Ehd1* under 10-h photoperiod conditions (Nemoto *et al.*, 2016). To ascertain whether *Hd16* is involved in its activation, we compared the *Hd16* and *Hd1* alleles in NILs of Koshihikari (Fig. 6A) and found that *Hd16* can enhance *Hd1* activity under 10-h photoperiod conditions. *Hd6* slightly represses *Ehd1* expression under 13.5-h photoperiod conditions (Fig. 4, right panel).

The natural variation of *Hd6* and *Hd16* were investigated in cultivars of *temperate japonica*, a rice subspecies adapted to northern temperate regions with longer photoperiods in the summer. Selected natural variation in the *Hd6* and *Hd16* genes have been identified only in *temperate japonica* cultivars grown in Japan; for example, Nipponbare, a typical such cultivar, has the defective allele of *Hd6*, whilst Koshihikari, the background *temperate japonica* cultivar in this work, has the defective alleles of both the *Hd6* and *Hd16* genes. The adaptation to northern temperate regions implies that such natural variation are due to relatively new mutations that could be utilized in rice breeding, and it is likely that this natural variation has been preferred in the temperate cultivation areas, such as the Japanese mainland. This adaptation is consistent with the fact that *Hd16* or *Hd6* influenced the gene expression of *Ehd1*, *Hd3a*, and *RFT1* only under 13- and 13.5-h photoperiods (Figs 3D, 7, and Supplementary Fig. S4). In addition, the effect of both *Hd16* and *Hd6* together on *Hd3a* and *RFT1* expression was greatly enhanced at 12-, 12.5-, 13-, and 13.5-h, but not at 10- and 14.5-h photoperiods (Fig. 3D). Thus, these genetic components can confer fine-tuning of the critical day length and may increase the ecological niches within latitude clines. Therefore, rice breeders may potentially aim to select the appropriate combination of *Hd16* and *Hd6* alleles for flowering time to fit with their target cultivation areas when they develop new cultivars of *temperate japonica*.

### *Hd16* and *Hd6* might function as a floral promoter under 10-h photoperiod conditions

The flowering-time phenotypes of rice grown under field conditions at Tsukuba, Japan, could be mimicked under the longer photoperiod conditions applied in artificially controlled growth chambers. As *Hd16* and *Hd6* were first isolated as floral repressors under natural field conditions after QTL cloning, this study investigated their effects on flowering phenotypes and related gene expression under artificially controlled 10-h photoperiod conditions in growth chambers, which simulate typical SD conditions.

Slightly early-flowering phenotypes were exhibited when both *Hd16* and *Hd6* alleles were functional (Fig. 3B). Thus, *Hd16* and *Hd6* seemed to act as a floral promoter under 10-h photoperiod conditions. Consistently, we found that both *Hd16* and *Hd6* together could activate *Ehd1* and *Hd3a* (weak activation) at night (see Supplementary Fig. S3) under 10-h photoperiod conditions. Under longer photoperiods, such as 12.5 h, the promotion of flowering disappeared (Fig. 3B). In addition, *Hd16* could activate *Ehd1*, but not *Hd3a* or *RFT1*, during the early night period under 12.5-h photoperiod conditions regardless of the *Hd6* genotype (Fig. 4 left panel). These results imply that *Hd16* may work as an activator of rice flowering in the presence of relatively short photoperiods. Taking these findings together, *Hd16* and *Hd6* might function as a floral promoter under SD conditions, although *RFT1* expression in the morning was not clearly affected by *Hd16* and *Hd6* (Fig. 3D). The promotion of activity of related genes by *Hd16* was more robust than that of *Hd6* under SD conditions; however, earlier flowering and greater *Hd3a* expression were observed only with both *Hd16* and *Hd6* functional alleles.

### *Hd16* assists *Hd1* activity under both LD and SD conditions

Previously, we reported that *Hd1* activates *Ehd1* regardless of *Ghd7* genotype during the night under 10-h photoperiod conditions (Nemoto *et al.*, 2016). In addition, both *Hd16* and *Hd6* together may have a bifunctional action to control flowering time, similar to *Hd1*. These results led us to examine whether *Ehd1* activation by either *Hd1* or *Hd16* occurred independently of each other. The results showed that *Hd16* and *Hd1* work together to activate *Ehd1* under 10-h photoperiod conditions (Fig. 7B). Furthermore, *Hd1* activated *Hd3a* expression after lights-off under 10-h photoperiod conditions (see Supplementary Fig. S5). Activation of *Hd3a* expression was also enhanced by functional *Hd16* (Supplementary Fig. S5). These results suggest that *Hd16* can enhance *Hd3a* expression at night under 10-h photoperiod conditions through the function of *Hd1* (Supplementary Figs S4 and S5).

Under 13.5-h photoperiod conditions, *Hd1* repressed *Ehd1* and *Hd3a* in the morning (Fig. 6B). Interestingly, *Hd16* also repressed *Hd3a* expression, which required the functional *Hd1* allele, but not *Ehd1* (Fig. 6B). On the other hand, *Hd16* repressed *Ehd1* expression with the defective *hd1* allele (Fig. 6B). These results implied that *Ehd1* and *Hd3a* repression by *Hd16* might occur through Ghd7 phosphorylation. Notably, rHd16 can phosphorylate rGhd7 *in vitro* (Hori *et al.*, 2013), and *Ghd7* can interact with *Hd1* to repress *Ehd1* and *Hd3a* (Nemoto *et al.*, 2016). Taking these findings together, *Hd16* seems to support the functional enhancement of *Hd1* activity, which can change between activator and repressor under certain photoperiod conditions. In addition, rHd1 is not phosphorylated by rHd16 and rHd6 *in vitro* (Ogiso *et al.*, 2010; Hori *et al.*, 2013). However, *Hd16* and *Hd6* interact with the *Hd2*/*OsPRR37* protein *in vivo*, and different regions of *Hd2*/*OsPRR37* are phosphorylated (Kwon *et al.*, 2015); rHd16 phosphorylates rGhd7 *in vitro* (Hori *et al.*, 2013). The diurnal expression patterns of *Hd2*/*OsPRR37* and *Ghd7* were not affected by *Hd16* and *Hd6* under 12.5- and 13.5-h photoperiod conditions (Fig. 4). These results imply that the activity of *Hd2/OsPRR37* and *Ghd7* gene products might be regulated by post-translational modification by *Hd16* and *Hd6* together to control florigen genes in rice, partially through the physical interaction between *Hd1* and *Ghd7* gene products (Nemoto *et al.*, 2016). *Hd2* functioned to delay flowering under the 14.5-h photoperiod and caused significantly later flowering when the functional allele of *Hd16* was present (Hori *et al.*, 2013). Future studies are required to determine whether Hd1 can interact physically with Hd2 *in vivo* to control downstream genes such as *Ehd1*, *Hd3a*, and *RFT1*.

## Supplementary data

Supplementary data are available at *JXB* online.

Fig. S1. Graphical representations of the genotypes of the NILs *Hd16Hd6* and *hd16Hd6* with SSR markers.

Fig. S2. Alignments of *Hd16* and *Hd6* coding sequences and deduced amino acid sequences.

Fig. S3. The non-functional allele of *hd16* showed higher repression of *Ehd1* expression than the functional *Hd16*, independent of *Hd6* function, under a 10-h photoperiod.

Fig. S4. Illustration of *Hd3a* expression regulated by *Hd6* and *Hd16* in the morning and at night under various photoperiod conditions.

Fig. S5. Expression of *Hd3a* in NILs at night under a 10-h photoperiod.

Supplementary Figures S1-S5

## Author contributions

TI conceived and designed all the experiments in this work; YN performed most of the experiments; KH prepared a series of NIL lines; YN and TI wrote the manuscript and KH revised it.
