# Supplementary material for: Fine-tuning of the setting of critical day length by two casein kinases in rice photoperiodic flowering
Source: J Exp Bot. 2017 Dec 10;69(3):553–65. doi: 10.1093/jxb/erx412 (PMC5853454; doi:10.1093/jxb/erx412)
Supplement: Supplementary Figures S1-S5 [file erx412_suppl_supplementary_figures_s1-s5.pdf]

Supplementary data

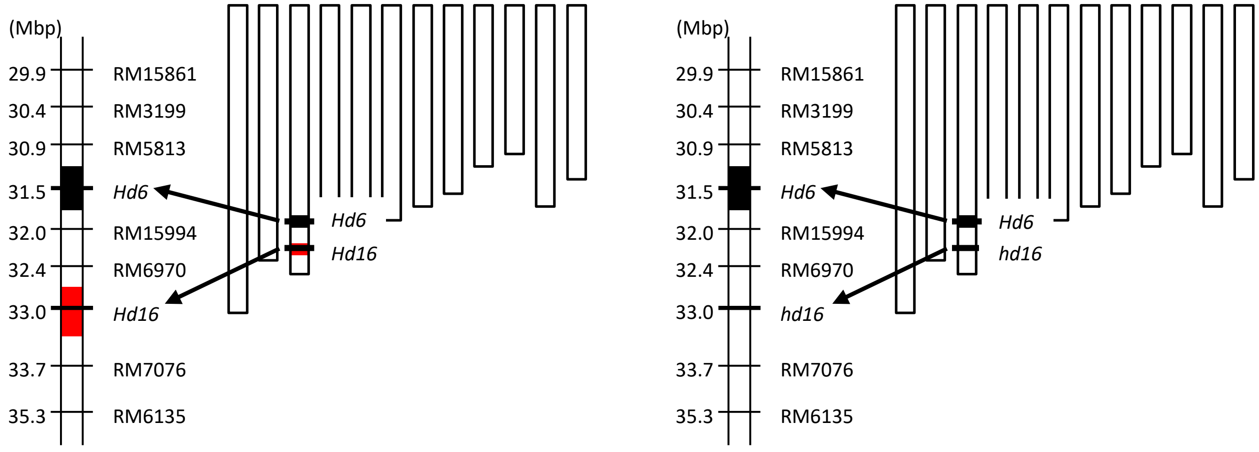

Fig. S1. Graphical representations of the genotypes of the NIL(*Hd16*, *Hd6*) and NIL (*hd16*, *Hd6*) with SSR markers. The 12 vertical bars represent the rice chromosomes. White, black and red bars indicate the Koshihikari, Kasalath and Nipponbare chromosome regions, respectively. Arrows indicate the positions of the *Hd6* and *Hd16* loci.

A

```
Nona Bokra       1 ATGCCAGAGTTGCGGGGTGGTGTTTGGAGAGCTCGTCTGAGGTCCAAGAAGGTTTACGACGTCCAGGACGCAGATCCAGCTGCGAGTCCG     90
Nipponbare       1 ATGCCAGAGTTGCGGGGTGGTGTTTGGAGAGCTCGTCTGAGGTCCAAGAAGGTTTACGACGTCCAGGACGCAGATCCAGCTGCGAGTCCG     90
Koshihikari      1 ATGCCAGAGTTGCGGGGTGGTGTTTGGAGAGCTCGTCTGAGGTCCAAGAAGGTTTACGACGTCCAGGACGCAGATCCAGCTGCGAGTCCG     90
                   ******************************************************************************************

Nona Bokra      91 GTGTCGCCAGCTCCGCGGGGGAGGACTGGAAGGCGCGGTGGTGCTGCGGCTGGCAGAGGCAACAAGACAGTTGCTGAGGGAGGAGGGAGG    180
Nipponbare      91 GTGTCGCCAGCTCCGCGGGGGAGGACTGGAAGGCGCGGCGGTGCTGCGGCTGGCAGAGGCAACAAGACAGTTGCTGAGGGAGGAGGGAGG    180
Koshihikari     91 GTGTCGCCAGCTCCGCGGGGGAGGACTGGAAGGCGCGGCGGTGCTGCGGCTGGCAGAGGCAACAAGACAGTTGCTGAGGGAGGAGGGAGG    180
                   **************************************.***************************************************

Nona Bokra     181 AAAGCTCTGAAGCCTAGAGGAAAGGGGTGCAGGGCTGTTGATTTGTGTGAGGATCAACCTTGCAAGGACCTCCCTGAAGGTATTGCTAGA    270
Nipponbare     181 AAAGCTCTGAAGCCTAGAGGAAAGGGGTGCAGGGCTGTTGATTTGTGTAAGGATCAACCTTGCAAGGACCTCCCTGAAGTTATTGCTAGA    270
Koshihikari    181 AAAGCTCTGAAGCCTAGAGGAAAGGGGTGCAGGGCTGTTGATTTGTGTAAGGATCAACCTTGCAAGGACCTCCCTGAAGTAATTGCTAGA    270
                   ************************************************.******************************..*********

Nona Bokra     271 AAGGCGGTTACCGGCAAAGCCCAGGAGGACCTTGGTTTGAACAAGGTAGCTGACAGAGCTGCGAACTTGATGATGGACGGCGAAAGTGGA    360
Nipponbare     271 AAGGCGGTTACCGGCAAAGCCCAGGAGGACCTTGGTTTGAACAAGGTAGCTGACAGAGCTGCGAACTTGATGATGGACGGCGAAAGTGGA    360
Koshihikari    271 AAGGCGGTTACCGGCAAAGCCCAGGAGGACCTTGGTTTGAACAAGGTAGCTGACAGAGCTGCGAACTTGATGATGGACGGCGAAAGTGGA    360
                   ******************************************************************************************

Nona Bokra     361 GACAAATTTGCTGCGGCAGAAGATGAATCCACTACAACACCAGTTCCTGAGCGGGTTCAAGTAGGCAATTCCCCAGAGTATATAACTGAT    450
Nipponbare     361 GACAAATTTGCTGCGGCAGAAGATGAATCCACTACAACACCAGTTCCTGAGCGGGTTCAAGTAGGCAATTCCCCAGAGTATATAACTGAT    450
Koshihikari    361 GACAAATTTGCTGCGGCAGAAGATGAATCCACTACAACACCAGTTCCTGAGCGGGTTCAAGTAGGCAATTCCCCAGAGTATATAACTGAT    450
                   ******************************************************************************************

Nona Bokra     451 AGGAAGTTGGGTAAAGGTGGATTTGGTCAGGTCTATGTTGGTCGAAGAGTATCTGGTGGAGGTTCTCGCACGGGTCCAGATGCGCAAGAG    540
Nipponbare     451 AGGAAGTTGGGTAAAGGTGGATTTGGTCAGGTCTATGTTGGTCGAAGAGTATCTGGTGGAGGTTCTCGCACGGGTCCAGATGCGCAAGAG    540
Koshihikari    451 AGGAAGTTGGGTAAAGGTGGATTTGGTCAGGTCTATGTTGGTCGAAGAGTATCTGGTGGAGGTTCTCGCACGGGTCCAGATGCGCAAGAG    540
                   ******************************************************************************************

Nona Bokra     541 GTTGCGCTTAAATTTGAGCACCGAAGCAGTAAAGGATGTAACTATGGCCCTCCATACGAGTGGCAGGTTTACCACACTCTCAATGGTTGT    630
Nipponbare     541 GTTGCGCTTAAATTTGAGCACCGAAGCAGTAAAGGATGTAACTATGGCCCTCCATACGAGTGGCAGGTTTACCACACTCTCAATGGTTGT    630
Koshihikari    541 GTTGCGCTTAAATTTGAGCACCGAAGCAGTAAAGGATGTAACTATGGCCCTCCATACGAGTGGCAGGTTTACCACACTCTCAATGGTTGT    630
                   ******************************************************************************************

Nona Bokra     631 TATGGCATACCATCGGTCCACTATAAGGGTCGTTTGGGAGACTACTACATTCTTGTAATGGATATGCTTGGTCCCAGCCTCTGGGATGTG    720
Nipponbare     631 TATGGCATACCATCAGTCCACTATAAGGGTCGTTTGGGAGACTACTACATTCTTGTAATGGATATGCTTGGTCCCAGCCTCTGGGATGTG    720
Koshihikari    631 TATGGCATACCATCAGTCCACTATAAGGGTCGTTTGGGAGACTACTACATTCTTGTAATGGATATGCTTGGTCCCAGCCTCTGGGATGTG    720
                   **************.***************************************************************************

Nona Bokra     721 TGGAATTCAGTGGGACAGGCGATGTCTGCCCATATGGTTGCTTGCATTGCTGTGGAAGCGATATCAATTCTTGAGAAGCTTCACTCTAAA    810
Nipponbare     721 TGGAATTCAGTGGGACAGGCGATGTCTGCCCATATGGTTGCTTGCATTGCTGTGGAAGCGATATCAATTCTTGAGAAGCTTCACTCTAAA    810
Koshihikari    721 TGGAATTCAGTGGGACAGGCGATGTCTGCCCATATGGTTGCTTGCATTGCTGTGGAAGCGATATCAATTCTTGAGAAGCTTCACTCTAAA    810
                   ******************************************************************************************

Nona Bokra     811 GGGTTTGTACATGGTGATGTCAAACCAGAGAATTTTTTGCTTGGTCATCCTGGGTCAGTTGATGAGAAGAAGCTTTTCCTGATTGATCTT    900
Nipponbare     811 GGGTTTGTACATGGTGATGTCAAACCAGAGAATTTTTTGCTTGGTCATCCTGGGTCAGTTGATGAGAAGAAGCTTTTCCTGATTGATCTT    900
Koshihikari    811 GGGTTTGTACATGGTGATGTCAAACCAGAGAATTTTTTGCTTGGTCATCCTGGGTCAGTTGATGAGAAGAAGCTTTTCCTGATTGATCTT    900
                   ******************************************************************************************

Nona Bokra     901 GGTTTAGCATCCAGGTGGAAAGAAGCATCATCTGGTCAGCATGTTGACTATGATCAGAGGCCAGATGTCTTTAGGGGAACAATTAGATAC    990
Nipponbare     901 GGTTTAGCATCCAGGTGGAAAGAAGCATCATCTGGTCAGCATGTTGACTATGATCAGAGGCCAGATGTCTTTAGGGGAACAATTAGATAC    990
Koshihikari    901 GGTTTAGCATCCAGGTGGAAAGAAGCATCATCTGGTCAGCATGTTGACTATGATCAGAGGCCAGATGTCTTTAGGGGAACAATTAGATAC    990
                   ******************************************************************************************

Nona Bokra     991 GCTAGCGTCCATGCCCACTTAGGTCGTACAGGTAGCAGGAGGGATGATTTAGAGTCACTGGCTTACACCCTAATCTTTTTAATAAGAGGG   1080
Nipponbare     991 GCTAGCGTCCATGCCCACTTAGGTCGTACAGGTAGCAGGAGGGATGATTTAGAGTCACTGGCTTACACCCTAATCTTTTTAATAAGAGGG   1080
Koshihikari    991 ACTAGCGTCCATGCCCACTTAGGTCGTACAGGTAGCAGGAGGGATGATTTAGAGTCACTGGCTTACACCCTAATCTTTTTAATAAGAGGG   1080
                   .*****************************************************************************************

Nona Bokra    1081 AGATTACCTTGGCAAGGGTATCAGGGAGATAACAAGAGTTTTCTTGTTTGTAAGAAGAAAATGGCTACTTCACCAGAGTTGCTGTGTTGC   1170
Nipponbare    1081 AGATTACCTTGGCAAGGGTATCAGGGAGATAACAAGAGTTTTCTTGTTTGTAAGAAGAAAATGGCTACTTCACCAGAGTTGCTGTGTTGC   1170
Koshihikari   1081 AGATTACCTTGGCAAGGGTATCAGGGAGATAACAAGAGTTTTCTTGTTTGTAAGAAGAAAATGGCTACTTCACCAGAGTTGCTGTGTTGC   1170
                   ******************************************************************************************

Nona Bokra    1171 TTCTGTCCAGCTCCGTTCAAACATTTTCTAGAGATGGTCACTAACATGAAATTTGACGAAGAGCCAAACTATCCAAAACTTATTTCTCTC   1260
Nipponbare    1171 TTCTGTCCAGCTCCGTTCAAACATTTTCTAGAGATGGTCACTAACATGAAATTTGACGAAGAGCCAAACTATCCAAAACTTATTTCTCTC   1260
Koshihikari   1171 TTCTGTCCAGCTCCGTTCAAACATTTTCTAGAGATGGTCACTAACATGAAATTTGACGAAGAGCCAAACTATCCAAAACTTATTTCTCTC   1260
                   ******************************************************************************************

Nona Bokra    1261 TTTGATGGTTTGATTGAAGGGCCTGCTTCAAGGCCCATCAGAATTGATGGAGCTCTGAAGGTTGGGCAAAAACGTGGAAGAATGGTTGTA   1350
Nipponbare    1261 TTTGATGGTTTGATTGAAGGGCCTGCTTCAAGGCCCATCAGAATTGATGGAGCTCTGAAGGTTGGGCAAAAACGTGGAAGAATGGTTGTA   1350
Koshihikari   1261 TTTGATGGTTTGATTGAAGGGCCTGCTTCAAGGCCCATCAGAATTGATGGAGCTCTGAAGGTTGGGCAAAAACGTGGAAGAATGGTTGTA   1350
                   ******************************************************************************************

Nona Bokra    1351 AATCTTGACGATGATGAACAGCCCAAGAAGAAAGTTAGGTTGGGAAGCCCAGCAACTCAATGGATCTCAGTTTATAATGCTAGGCGGCCC   1440
Nipponbare    1351 AATCTTGACGATGATGAACAGCCCAAGAAGAAAGTTAGGTTGGGAAGCCCAGCAACTCAATGGATCTCAGTTTATAATGCTAGGCGGCCC   1440
Koshihikari   1351 AATCTTGACGATGATGAACAGCCCAAGAAGAAAGTTAGGTTGGGAAGCCCAGCAACTCAATGGATCTCAGTTTATAATGCTAGGCGGCCC   1440
                   ******************************************************************************************

Nona Bokra    1441 ATGAAGCAGAGATATCACTATAATGTTGCTGATTCAAGGCTGCATCAGCATATAGAAAAAGGCAATGAAGATGGGCTGTACATTAGTTGT   1530
Nipponbare    1441 ATGAAGCAGAGATATCACTATAATGTTGCTGATTCAAGGCTGCATCAGCATATAGAAAAAGGCAATGAAGATGGGCTGTACATTAGTTGT   1530
Koshihikari   1441 ATGAAGCAGAGATATCACTATAATGTTGCTGATTCAAGGCTGCATCAGCATATAGAAAAAGGCAATGAAGATGGGCTGTACATTAGTTGT   1530
                   ******************************************************************************************

Nona Bokra    1531 GTCTCTTCTTCCGCAAATTTTTGGGCTCTCATAATGGATGCTGGGACTGGCTTTTGTTCCCAAGTTTATGAGCTTTCACAAGTGTTTCTG   1620
Nipponbare    1531 GTCTCTTCTTCCGCAAATTTTTGGGCTCTCATAATGGATGCTGGGACTGGCTTTTGTTCCCAAGTTTATGAGCTTTCACAAGTGTTTCTG   1620
Koshihikari   1531 GTCTCTTCTTCCGCAAATTTTTGGGCTCTCATAATGGATGCTGGGACTGGCTTTTGTTCCCAAGTTTATGAGCTTTCACAAGTGTTTCTG   1620
                   ******************************************************************************************

Nona Bokra    1621 CACAAGGATTGGATTATGGAGCAGTGGGAGAAGAATTATTACATAACGGCAATAGCAGGAGCAACCAATGGAAGCTCATTGGTTGTAATG   1710
Nipponbare    1621 CACAAGGATTGGATTATGGAGCAGTGGGAGAAGAATTATTACATAACGGCAATAGCAGGAGCAACCAATGGAAGCTCATTGGTTGTAATG   1710
Koshihikari   1621 CACAAGGATTGGATTATGGAGCAGTGGGAGAAGAATTATTACATAACGGCAATAGCAGGAGCAACCAATGGAAGCTCATTGGTTGTAATG   1710
                   ******************************************************************************************
```

```
Nona Bokra    1711 TCCAAAGGAACTCCATACACACAGCAGTCATACAAAGTCAGTGAATCCTTTCCTTACAAGTGGATTAACAAAAAGTGGAAAGAAGGTTTC   1800
Nipponbare    1711 TCCAAAGGAACTCCATACACACAGCAGTCATACAAAGTCAGTGAATCCTTTCCTTACAAGTGGATTAACAAAAAGTGGAAAGAAGGTTTC   1800
Koshihikari   1711 TCCAAAGGAACTCCATACACACAGCAGTCATACAAAGTCAGTGAATCCTTTCCTTACAAGTGGATTAACAAAAAGTGGAAAGAAGGTTTC   1800
                   ******************************************************************************************

Nona Bokra    1801 CATGTGACATCTATGGCTACTGCTGGAAACCGTTGGGGAGTTGTCATGTCAAGAAATGCAGGCTATTCCCATCAGGTGGTAGAGTTGGAC   1890
Nipponbare    1801 CATGTGACATCTATGGCTACTGCTGGAAACCGTTGGGGAGTTGTCATGTCAAGAAATGCAGGCTATTCCCATCAGGTGGTAGAGTTGGAC   1890
Koshihikari   1801 CATGTGACATCTATGGCTACTGCTGGAAACCGTTGGGGAGTTGTCATGTCAAGGAATGCAGGCTATTCCCATCAGGTGGTAGAGTTGGAC   1890
                   *****************************************************.************************************

Nona Bokra    1891 TTTCTATATCCAAGTGAAGGGATCCATCGGCGATGGGAGACAGGTTACAGAATAACTTCGACTGCAGCAACTCCTGACCAAGCTGCCTTC   1980
Nipponbare    1891 TTTCTATATCCAAGTGAAGGGATCCATCGGCGATGGGAGACAGGTTACAGAATAACTTCGACTGCAGCAACTCCTGACCAAGCTGCCTTC   1980
Koshihikari   1891 TTTCTATATCCAAGTGAAGGGATCCATCGGCGATGGGAGACAGGTTACAGAATAACTTCGACTGCAGCAACTCCTGACCAAGCTGCCTTC   1980
                   ******************************************************************************************

Nona Bokra    1981 ATCTTGAGCATACCAAAGAGGAAGCCAATGGACGAGACACAAGAAACTCTTCGAACTTCCTCCTTTCCCAGCAACCATGTCAAGGAAAAA   2070
Nipponbare    1981 ATCTTGAGCATACCAAAGAGGAAGCCAATGGACGAGACACAAGAAACTCTTCGAACTTCCTCCTTTCCCAGCAACCATGTCAAGGAAAAA   2070
Koshihikari   1981 ATCTTGAGCATACCAAAGAGGAAGCCAATGGACGAGACACAAGAAACTCTTCGAACTTCCTCCTTTCCCAGCAACCATGTCAAGGAAAAA   2070
                   ******************************************************************************************

Nona Bokra    2071 TGGTCAAAGAACCTATACATCGCTTCAATCTGCTACGGCCGGACCGTATGCTGA                                       2124
Nipponbare    2071 TGGTCAAAGAACCTATACATCGCTTCAATCTGCTACGGCCGGACCGTATGCTGA                                       2124
Koshihikari   2071 TGGTCAAAGAACCTATACATCGCTTCAATCTGCTACGGCCGGACCGTATGCTGA                                       2124
                   ******************************************************
```

B

```
Nona Bokra       1 MPELRGGVWRARLRSKKVYDVQDADPAASPVSPAPRGRTGRRGGAAAGRGNKTVAEGGGRKALKPRGKGCRAVDLCEDQPCKDLPEGIAR     90
Nipponbare       1 MPELRGGVWRARLRSKKVYDVQDADPAASPVSPAPRGRTGRRGGAAAGRGNKTVAEGGGRKALKPRGKGCRAVDLCKDQPCKDLPEVIAR     90
Koshihikari      1 MPELRGGVWRARLRSKKVYDVQDADPAASPVSPAPRGRTGRRGGAAAGRGNKTVAEGGGRKALKPRGKGCRAVDLCKDQPCKDLPEVIAR     90
                   ****************************************************************************.*********.***

Nona Bokra      91 KAVTGKAQEDLGLNKVADRAANLMMDGESGDKFAAAEDESTTTPVPERVQVGNSPEYITDRKLGKGGFGQVYVGRRVSGGGSRTGPDAQE    180
Nipponbare      91 KAVTGKAQEDLGLNKVADRAANLMMDGESGDKFAAAEDESTTTPVPERVQVGNSPEYITDRKLGKGGFGQVYVGRRVSGGGSRTGPDAQE    180
Koshihikari     91 KAVTGKAQEDLGLNKVADRAANLMMDGESGDKFAAAEDESTTTPVPERVQVGNSPEYITDRKLGKGGFGQVYVGRRVSGGGSRTGPDAQE    180
                   ******************************************************************************************

Nona Bokra     181 VALKFEHRSSKGCNYGPPYEWQVYHTLNGCYGIPSVHYKGRLGDYYILVMDMLGPSLWDVWNSVGQAMSAHMVACIAVEAISILEKLHSK    270
Nipponbare     181 VALKFEHRSSKGCNYGPPYEWQVYHTLNGCYGIPSVHYKGRLGDYYILVMDMLGPSLWDVWNSVGQAMSAHMVACIAVEAISILEKLHSK    270
Koshihikari    181 VALKFEHRSSKGCNYGPPYEWQVYHTLNGCYGIPSVHYKGRLGDYYILVMDMLGPSLWDVWNSVGQAMSAHMVACIAVEAISILEKLHSK    270
                   ******************************************************************************************

Nona Bokra     271 GFVHGDVKPENFLLGHPGSVDEKKLFLIDLGLASRWKEASSGQHVDYDQRPDVFRGTIRYASVHAHLGRTGSRRDDLESLAYTLIFLIRG    360
Nipponbare     271 GFVHGDVKPENFLLGHPGSVDEKKLFLIDLGLASRWKEASSGQHVDYDQRPDVFRGTIRYASVHAHLGRTGSRRDDLESLAYTLIFLIRG    360
Koshihikari    271 GFVHGDVKPENFLLGHPGSVDEKKLFLIDLGLASRWKEASSGQHVDYDQRPDVFRGTIRYTSVHAHLGRTGSRRDDLESLAYTLIFLIRG    360
                   ************************************************************.*****************************

Nona Bokra     361 RLPWQGYQGDNKSFLVCKKKMATSPELLCCFCPAPFKHFLEMVTNMKFDEEPNYPKLISLFDGLIEGPASRPIRIDGALKVGQKRGRMVV    450
Nipponbare     361 RLPWQGYQGDNKSFLVCKKKMATSPELLCCFCPAPFKHFLEMVTNMKFDEEPNYPKLISLFDGLIEGPASRPIRIDGALKVGQKRGRMVV    450
Koshihikari    361 RLPWQGYQGDNKSFLVCKKKMATSPELLCCFCPAPFKHFLEMVTNMKFDEEPNYPKLISLFDGLIEGPASRPIRIDGALKVGQKRGRMVV    450
                   ******************************************************************************************

Nona Bokra     451 NLDDDEQPKKKVRLGSPATQWISVYNARRPMKQRYHYNVADSRLHQHIEKGNEDGLYISCVSSSANFWALIMDAGTGFCSQVYELSQVFL    540
Nipponbare     451 NLDDDEQPKKKVRLGSPATQWISVYNARRPMKQRYHYNVADSRLHQHIEKGNEDGLYISCVSSSANFWALIMDAGTGFCSQVYELSQVFL    540
Koshihikari    451 NLDDDEQPKKKVRLGSPATQWISVYNARRPMKQRYHYNVADSRLHQHIEKGNEDGLYISCVSSSANFWALIMDAGTGFCSQVYELSQVFL    540
                   ******************************************************************************************

Nona Bokra     541 HKDWIMEQWEKNYYITAIAGATNGSSLVVMSKGTPYTQQSYKVSESFPYKWINKKWKEGFHVTSMATAGNRWGVVMSRNAGYSHQVVELD    630
Nipponbare     541 HKDWIMEQWEKNYYITAIAGATNGSSLVVMSKGTPYTQQSYKVSESFPYKWINKKWKEGFHVTSMATAGNRWGVVMSRNAGYSHQVVELD    630
Koshihikari    541 HKDWIMEQWEKNYYITAIAGATNGSSLVVMSKGTPYTQQSYKVSESFPYKWINKKWKEGFHVTSMATAGNRWGVVMSRNAGYSHQVVELD    630
                   ******************************************************************************************

Nona Bokra     631 FLYPSEGIHRRWETGYRITSTAATPDQAAFILSIPKRKPMDETQETLRTSSFPSNHVKEKWSKNLYIASICYGRTVC                 707
Nipponbare     631 FLYPSEGIHRRWETGYRITSTAATPDQAAFILSIPKRKPMDETQETLRTSSFPSNHVKEKWSKNLYIASICYGRTVC                 707
Koshihikari    631 FLYPSEGIHRRWETGYRITSTAATPDQAAFILSIPKRKPMDETQETLRTSSFPSNHVKEKWSKNLYIASICYGRTVC                 707
                   *****************************************************************************
```

C

```
Nona Bokra     1 ATGTCGAAGGCGAGGGTCTACGCCGACGTCAACGTGCTGCGCCCCAAGGAGTACTGGGACTACGAGGCGCTCACCGTTCAATGGGGTGAG     90
Kasalath       1 ATGTCGAAGGCGAGGGTCTACGCCGACGTCAACGTGCTGCGCCCCAAGGAGTACTGGGACTACGAGGCGCTCACCGTTCAATGGGGTGAG     90
                 ******************************************************************************************

Nona Bokra    91 CAGGATGACTATGAAGTTGTCAGGAAAGTTGGAAGAGGTAAATATAGTGAAGTCTTTGAAGGCATCAATGTTAACAACAATGAGAAATGC    180
Kasalath      91 CAGGATGACTATGAAGTTGTCAGGAAAGTTGGAAGAGGTAAATATAGTGAAGTCTTTGAAGGCATCAATGTTAACAACAATGAGAAATGC    180
                 ******************************************************************************************

Nona Bokra   181 ATCATCAAGATACTCAAGCCTGTGAAGAAAAAGAAGATCAAAAGGGAGATTAAAATACTTCAGAATCTTTGTGGAGGTCCAAACATTGTG    270
Kasalath     181 ATCATCAAGATACTCAAGCCTGTGAAGAAAAAGAAGATCAAAAGGGAGATTAAAATACTTCAGAATCTTTGTGGAGGTCCAAACATTGTG    270
                 ******************************************************************************************

Nona Bokra   271 AAGCTTCTTGATATTGTCAGAGATCAACATTCTAAGACTCCTAGCTTGATCTTTGAATATGTCAACAATACAGACTTCAAAGTGCTGTAC    360
Kasalath     271 AAGCTTCTTGATATTGTCAGAGATCAACATTCTAAGACTCCTAGCTTGATCTTTGAATATGTCAACAATACAGACTTCAAAGTGCTGTAC    360
                 ******************************************************************************************

Nona Bokra   361 CCCACGTTGACAGATTATGATATCCGCTACTACATATATGAGCTACTCAAGGCATTAGACTACTGCCATTCACAAGGCATTATGCATCGA    450
Kasalath     361 CCCACGTTGACAGATTATGATATCCGCTACTACATATATGAGCTACTCAAGGCATTAGACTACTGCCATTCACAAGGCATTATGCATCGA    450
                 ******************************************************************************************

Nona Bokra   451 GATGTCAAGCCCCACAATGTTATGATAGATCATGAGCTCCGAAAACTTCGATTGATAGACTGGGGCCTGGCTGAGTTCTATCATCCAGGG    540
Kasalath     451 GATGTCAAGCCCCACAATGTTATGATAGATCATGAGCTCCGAAAACTTCGATTGATAGACTGGGGCCTGGCTGAGTTCTATCATCCAGGG    540
                 ******************************************************************************************

Nona Bokra   541 AAGGAATATAATGTTCGTGTTGCTTCAAGGTATTTCAAGGGGCCTGAGCTTCTTGTTGATTTGCAAGATTATGATTATTCTTTGGACATG    630
Kasalath     541 AAGGAATATAATGTTCGTGTTGCTTCAAGGTATTTCAAGGGGCCTGAGCTTCTTGTTGATTTGCAAGATTATGATTATTCTTTGGACATG    630
                 ******************************************************************************************

Nona Bokra   631 TGGAGCCTTGGTTGCATGTTTGCTGGGATGATATTCCGCAAGGAGCCATTCTTCTATGGTCATGATAACCATGATCAACTTGTCAAGATC    720
Kasalath     631 TGGAGCCTTGGTTGCATGTTTGCTGGGATGATATTCCGCAAGGAGCCATTCTTCTATGGTCATGATAACCATGATCAACTTGTCAAGATC    720
                 ******************************************************************************************

Nona Bokra   721 GCAAAGGTACTTGGAACAGAAGCACTAAATGCTTATTTGAACAAGTACCATATTGAGCTTGATCCTCAGCTTGAAGCTCTTGTTGGGAGG    810
Kasalath     721 GCAAAGGTACTTGGAACAGAAGCACTAAATGCTTATTTGAACAAGTACCATATTGAGCTTGATCCTCAGCTTGAAGCTCTTGTTGGGAGG    810
                 ******************************************************************************************

Nona Bokra   811 CATAGTAGAAAACCATGGTCGAAATTCATTAATGCTGATAACCAACATCTAGTATCTCCTGAGGCTGTAGATTTTCTTGATAAGCTTCTA    900
Kasalath     811 CATAGTAGAAAACCATGGTCGAAATTCATTAATGCTGATAACCAACATCTAGTATCTCCTGAGGCTGTAGATTTTCTTGATAAGCTTCTA    900
                 ******************************************************************************************

Nona Bokra   901 CGTTATGATCACCAAGATAGGCTCACTGCACGTGAAGCTATGGCACATCCGTACTTCCTCCAAGTGAGAGCTGCAGAAAATAGCAGAGCA    990
Kasalath     901 CGTTATGATCACCAAGATAGGCTCACTGCACGTGAAGCTATGGCACATCCGTACTTCCTCCAAGTGAGAGCTGCAGAAAATAGCAGAGCA    990
                 ******************************************************************************************

Nona Bokra   991 CGACCACAATGA                                                                              1002
Kasalath     991 CGACCACAATGA                                                                              1002
                 ************
```

D

```
Nona Bokra     1 MSKARVYADVNVLRPKEYWDYEALTVQWGEQDDYEVVRKVGRGKYSEVFEGINVNNNEKCIIKILKPVKKKKIKREIKILQNLCGGPNIV     90
Kasalath       1 MSKARVYADVNVLRPKEYWDYEALTVQWGEQDDYEVVRKVGRGKYSEVFEGINVNNNEKCIIKILKPVKKKKIKREIKILQNLCGGPNIV     90
                 ******************************************************************************************

Nona Bokra    91 KLLDIVRDQHSKTPSLIFEYVNNTDFKVLYPTLTDYDIRYYIYELLKALDYCHSQGIMHRDVKPHNVMIDHELRKLRLIDWGLAEFYHPG    180
Kasalath      91 KLLDIVRDQHSKTPSLIFEYVNNTDFKVLYPTLTDYDIRYYIYELLKALDYCHSQGIMHRDVKPHNVMIDHELRKLRLIDWGLAEFYHPG    180
                 ******************************************************************************************

Nona Bokra   181 KEYNVRVASRYFKGPELLVDLQDYDYSLDMWSLGCMFAGMIFRKEPFFYGHDNHDQLVKIAKVLGTEALNAYLNKYHIELDPQLEALVGR    270
Kasalath     181 KEYNVRVASRYFKGPELLVDLQDYDYSLDMWSLGCMFAGMIFRKEPFFYGHDNHDQLVKIAKVLGTEALNAYLNKYHIELDPQLEALVGR    270
                 ******************************************************************************************

Nona Bokra   271 HSRKPWSKFINADNQHLVSPEAVDFLDKLLRYDHQDRLTAREAMAHPYFLQVRAAENSRARPQ                            333
Kasalath     271 HSRKPWSKFINADNQHLVSPEAVDFLDKLLRYDHQDRLTAREAMAHPYFLQVRAAENSRARPQ                            333
                 ***************************************************************
```

Fig. S2. Alignments of *Hd16* (A and B) and *Hd6* (C and D) coding sequences (CDS) and deduced amino acid sequences. *Hd16* CDS from Nona Bokra was compared with Nipponbare and Koshihikari CDSs (A). There were four or seven nucleotide differences between Nona Bokra and Nipponbare or Koshihikari, respectively (A). Among them, two SNPs, indicated in A with green box, showed nonsynonymous change (B with green box). Critical amino acid for CK1 function reported between Nipponbare and Koshihikari showed with red box. Nona Bokra was Nipponbare type of the amino acid (B). Alignments of *Hd6* CDS (C) and deduced amino acid sequences (D) of Nona Bokra and Kasalath. Nona Bokra showed the same coding sequence with Kasalath.

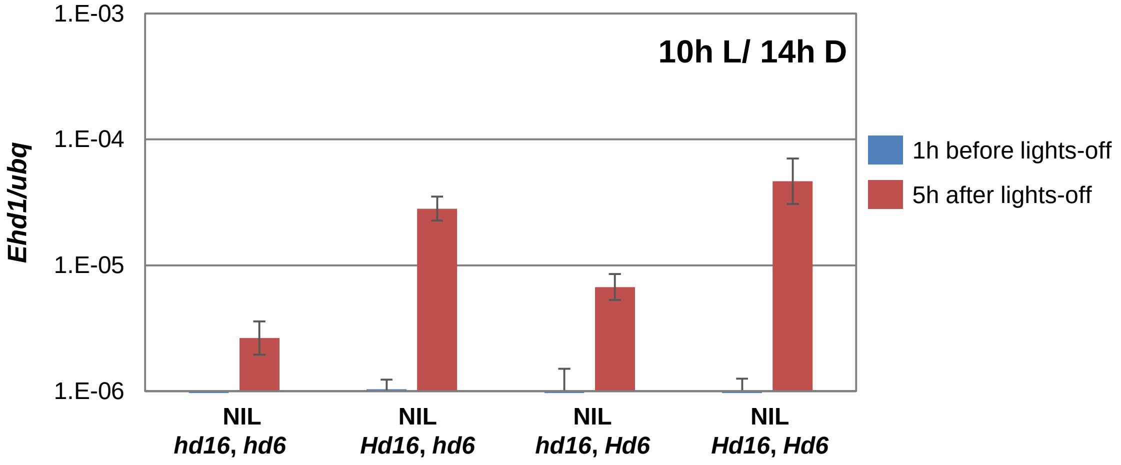

Figure S3. Non-functional allele of *hd16* showed higher repression of *Ehd1* expression than functional *Hd16*, independent of *Hd6* function, under 10h photoperiod. This result was similar to previous data under 12.5h photoperiod (Fig. 4 left panel).

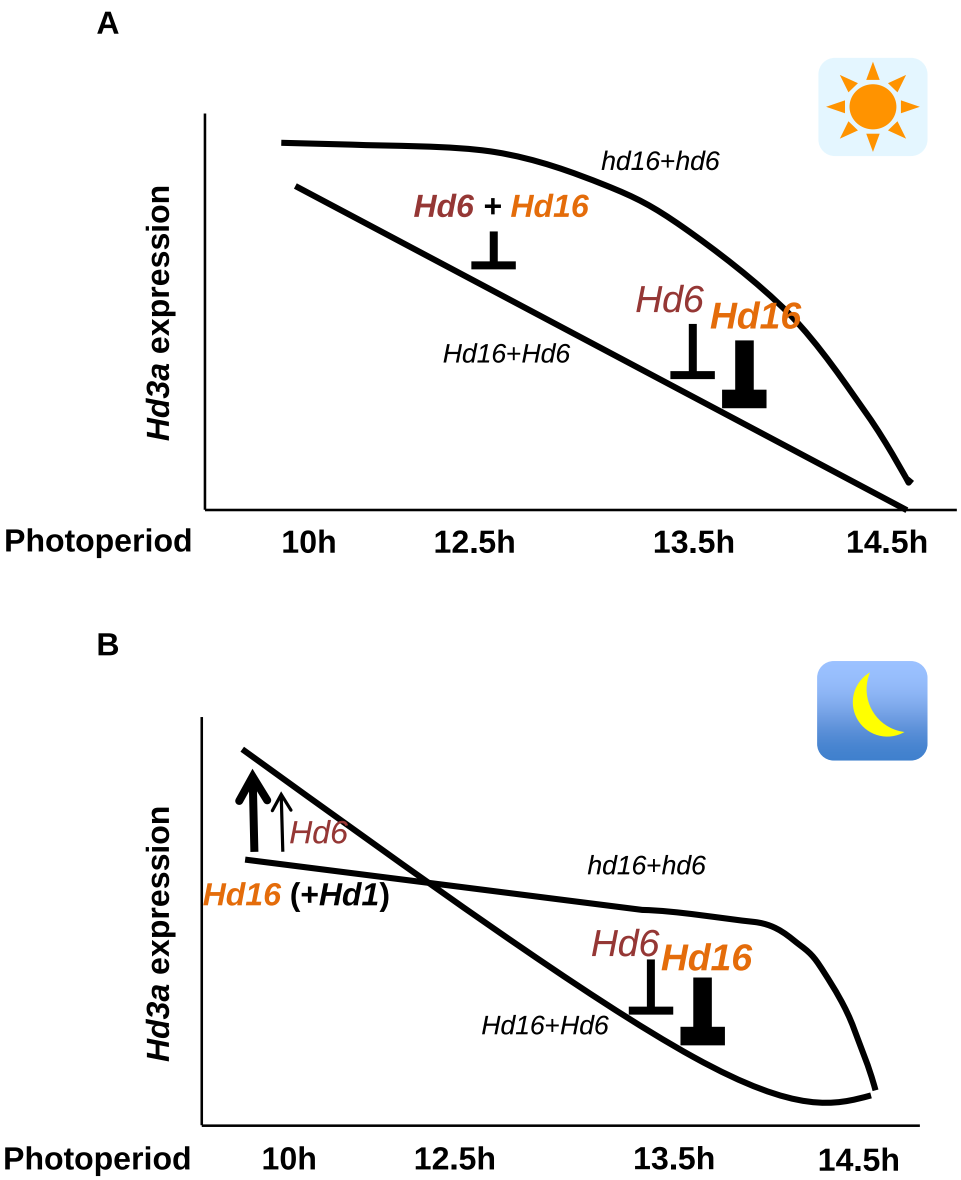

Figure S4. Illustration of *Hd3a* expression regulated by *Hd6* and *Hd16* during a day, in the morning (A) and at night (B) under various photoperiod conditions. Y axis indicated the expression level of *Hd3a* and X axis shows photoperiod. We compared functional (*Hd16* and *Hd6*) and non-functional (*hd16* and *hd6*) allele in the morning (A) and in the beginning of the night (B). The expression pattern in the morning showed that functional allele of *Hd16* and *Hd6* repressed *Hd3a* expression depending on increment of photoperiod. In the beginning of the night, functional allele of *Hd16* enhanced *Hd3a* activation by *Hd1*, like as *Ehd1* (Fig. 6A and S3). We also found that *Hd3a* was activated slightly when both allele, *Hd16 Hd6*, were functional.

10h L/ 14h D

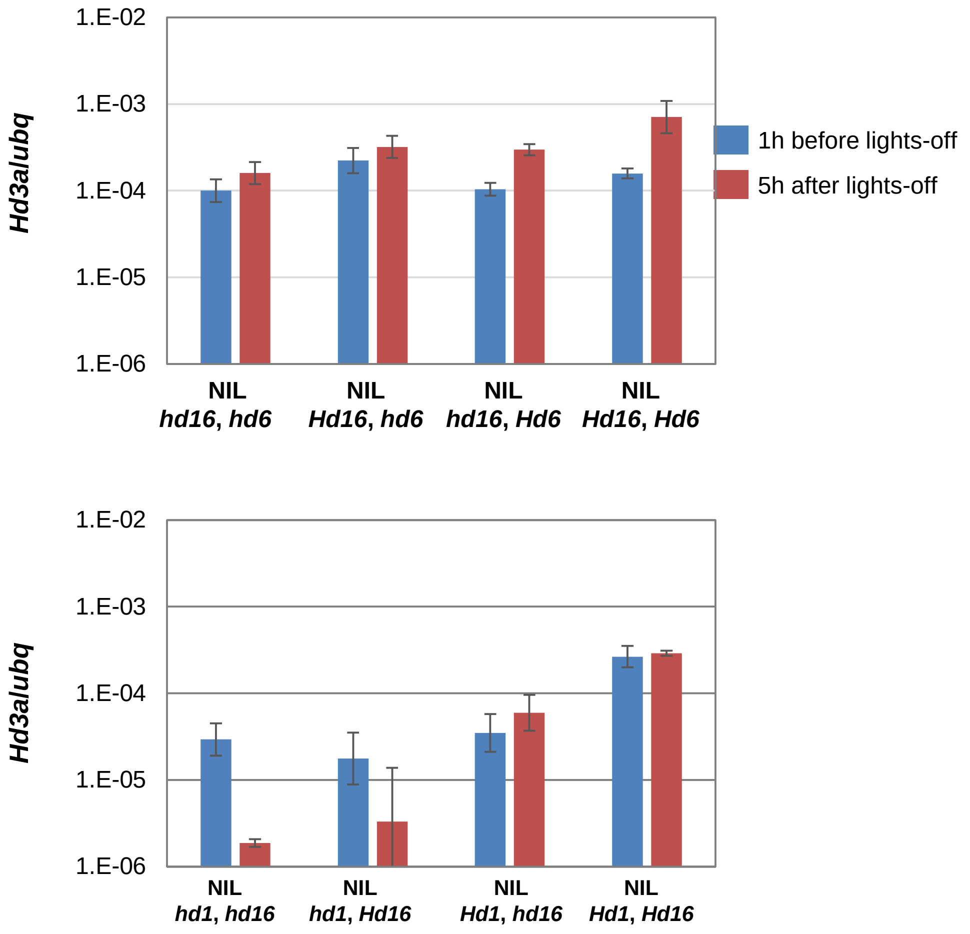

Figure S5. Expression of *Hd3a* in NILs at night under 10h photoperiod. *Hd3a* expression level were compared with functional and defective allele of *Hd16 Hd6* (upper graph) and *Hd16 Hd1* (lower graph). The same cDNA samples were used as Fig. S1 and 6A.
